# Human Claustrum Connections: Robust In Vivo Detection by DWI‐Based Tractography in Two Large Samples

**DOI:** 10.1002/hbm.70042

**Published:** 2024-10-13

**Authors:** Jil Wendt, Antonia Neubauer, Dennis M. Hedderich, Benita Schmitz‐Koep, Sevilay Ayyildiz, David Schinz, Rebecca Hippen, Marcel Daamen, Henning Boecker, Claus Zimmer, Dieter Wolke, Peter Bartmann, Christian Sorg, Aurore Menegaux

**Affiliations:** ^1^ Department of Diagnostic and Interventional Neuroradiology, School of Medicine and Health Technical University of Munich Munich Germany; ^2^ School of Medicine and Health, TUM‐NIC Neuroimaging Center Technical University of Munich Munich Germany; ^3^ Department of Diagnostic and Interventional Radiology, Clinical Functional Imaging Group University Hospital Bonn Bonn Germany; ^4^ Department of Psychology University of Warwick Coventry UK; ^5^ Warwick Medical School University of Warwick Coventry UK; ^6^ Department of Neonatology and Pediatric Intensive Care University Hospital Bonn Bonn Germany; ^7^ Department of Psychiatry, School of Medicine and Health Technical University of Munich Munich Germany

**Keywords:** claustrum, connectivity, DWI, HCP, magnetic resonance imaging, tractography

## Abstract

Despite substantial neuroscience research in the last decade revealing the claustrum's prominent role in mammalian forebrain organization, as evidenced by its extraordinarily widespread connectivity pattern, claustrum studies in humans are rare. This is particularly true for studies focusing on claustrum connections. Two primary reasons may account for this situation: First, the intricate anatomy of the human claustrum located between the external and extreme capsule hinders straightforward and reliable structural delineation. In addition, the few studies that used diffusion‐weighted‐imaging (DWI)‐based tractography could not clarify whether in vivo tractography consistently and reliably identifies claustrum connections in humans across different subjects, cohorts, imaging methods, and connectivity metrics. To address these issues, we combined a recently developed deep‐learning‐based claustrum segmentation tool with DWI‐based tractography in two large adult cohorts: 81 healthy young adults from the human connectome project and 81 further healthy young participants from the Bavarian longitudinal study. Tracts between the claustrum and 13 cortical and 9 subcortical regions were reconstructed in each subject using probabilistic tractography. Probabilistic group average maps and different connectivity metrics were generated to assess the claustrum's connectivity profile as well as consistency and replicability of tractography. We found, across individuals, cohorts, DWI‐protocols, and measures, consistent and replicable cortical and subcortical ipsi‐ and contralateral claustrum connections. This result demonstrates robust in vivo tractography of claustrum connections in humans, providing a base for further examinations of claustrum connectivity in health and disease.

## Graphical abstract

We investigated the connectivity of the human claustrum in two large cohorts using a deep‐learning‐based segmentation tool and diffusion‐weighted‐imaging (DWI)‐based tractography. Across individuals, cohorts, and DWI‐protocols, we found consistent and replicable ipsi‐ and contralateral connections with cortical and subcortical regions, laying the foundation for comprehensive investigations in health and disease.

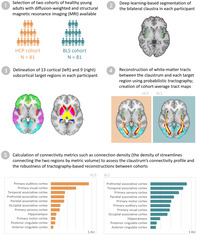

**Abbreviations**
BLS: Bavarian longitudinal study
CD: connection density
CP: connection probability
DWI: diffusion‐weighted imaging
HCP: human connectome project
MRI: magnetic resonance imaging
ROI: region of interest
T1w: T1‐weighted

## 1 Introduction

This study deals with the consistency and replicability of diffusion‐weighted imaging (DWI)‐based reconstruction of claustrum connections via probabilistic tractography in the human brain. It is motivated, on the one hand, by our rapidly increasing knowledge about the critical role of the claustrum in mammal forebrain physiology and function (Brown et al. 2017; Dillingham et al. 2017; Jackson, Smith, and Lee 2020), and on the other hand, by difficulties in imaging claustrum properties in the human brain due to its small and intricate form Nikolenko et al. (2021).

The claustrum is a small grey matter region composed of glutamatergic excitatory neurons and GABAergic interneurons (Bruguier et al. 2020), located between the external and extreme capsule and below the insular cortex in the brain (Jackson, Smith, and Lee 2020). It is present in all mammals (Kowiański et al. 1999), but its precise shape, location, and connections vary across species (Bruguier et al. 2020). Embryologically, the primordial claustrum's cells are initially formed in radial progenitor domains of the lateral pallium, temporally parallel with subplate cells of the dorsal pallium and with tangential migration between the two compartments, making the claustrum one of the very early formed forebrain structures (Watson and Puelles 2017; Smith et al. 2019; Bruguier et al. 2020). While it makes up only 0.25% of the cortex volume (Kowiański et al. 1999), it has been suggested to be the most densely connected forebrain structure by metric volume (Torgerson et al. 2015). Therefore, it is a highly interesting region to research.

The question of the claustrum's function(s) is far from being solved in a satisfactory way, particularly in humans. Due to its inaccessible size, shape, and location, few human cases of isolated lesions with loss of function that could serve to elucidate its purpose exist (Mathur 2014; Goll, Atlan, and Citri 2015). Since an early suggestion of a role in consciousness (Crick and Koch 2005; Smythies 2014), hypotheses have evolved considerably. Most recently, several studies identified the claustrum's involvement in a range of very different functions, for example in sleep and slow‐wave activity regulation (Renouard et al. 2015; Narikiyo et al. 2020; Norimoto et al. 2020), as a saliency detector (Remedios, Logothetis, and Kayser 2010; Smythies, Edelstein, and Ramachandran 2012a, 2012b), and in attentional load allocation (Atlan et al. 2018; White and Mathur 2018; White et al. 2020). It has been suggested that the basic physiology of claustrum‐cortex circuits, namely selective inhibitory suppression of the cortex, makes the claustrum suited for distributed but coordinated cortex control, which is in service of the aforementioned distinct functions (Jackson, Smith, and Lee 2020; Smith, Lee, and Jackson 2020). Additionally, anatomical changes have been described in several brain pathologies and disorders (Nikolenko et al. 2021), such as epilepsy (Zhang et al. 2001), autism (Wegiel et al. 2014), encephalitis (Ishii, Tsuji, and Tamaoka 2011), schizophrenia (Cascella et al. 2011), Parkinson's disease (Arrigo et al. 2019), and prematurity (Hedderich et al. 2021; Neubauer et al. 2023).

In order to understand the function of a brain structure, it is critical to investigate its connectivity pattern. Regarding claustrum connectivity, most of the current evidence available stems from studies in different mammals, mainly mice, using methods like viral tracer injection, high angular resolution diffusion imaging (HARDI), and functional MRI (fMRI). Across different studies and methods, there is strong evidence for widespread, reciprocal connections of the claustrum with the cortex (Edelstein and Denaro 2004; Smith and Alloway 2010; Smythies, Edelstein, and Ramachandran 2012b; Park, Tyszka, and Allman 2012; Atlan et al. 2017; White et al. 2017; Zingg et al. 2018; Wang et al. 2023), as well as many subcortical areas (Park, Tyszka, and Allman 2012; Wang et al. 2017; Zingg et al. 2018; Krimmel et al. 2019), which has previously been reviewed extensively (Mathur 2014; Smythies 2014; Torgerson and van Horn 2014; Dillingham et al. 2017; Jackson, Smith, and Lee 2020). Importantly for human research, the sum of findings from different animals shows marked differences across species (Jackson, Smith, and Lee 2020), suggesting that those findings may not be transferable to humans without reservations.

However, studying white matter connectivity in humans using in vivo techniques, especially in a structure as small and irregular as the claustrum, is challenging. Consequently, only a few studies in humans have been conducted, resorting to indirect methods of mapping white matter based on DWI. Briefly, by mapping the directionality of the displacement of water molecules within each voxel of the brain, large white matter pathways can be reconstructed, in an approach called DWI‐based tractography (Beaulieu 2002; Tournier, Mori, and Leemans 2011). Fernández‐Miranda et al. used a dual approach of tractography and fiber dissection in a small cohort, providing the first in vivo study of claustro‐cortical projections in humans and, importantly, showing the method's accuracy in direct comparison with fiber dissection (Fernández‐Miranda et al. 2008). Milardi et al. provided further evidence of large fiber tracts between the claustrum and most cortical areas, as well as of the existence of contralateral projections and interclaustral communications bundles (Milardi et al. 2015; Arrigo et al. 2017). Finally, a high‐yield theoretical graph‐based approach to whole‐brain tract connectivity patterns confirmed the widespread nature of connections and suggested the claustrum to have the highest density of connections per unit volume of any brain structure (Torgerson et al. 2015).

While these studies have shown the feasibility of visualizing some aspects of claustrum connectivity using DWI, and suggest the presence of extensive claustrum connectivity also in humans, it remains unclear if this indirect method is really able to accurately and—most importantly for the current study—robustly (i.e., consistently and replicably) reconstruct the extent of the claustrum's connections, amongst other open questions. Potential inexactitudes due to a small number of subjects as well as in high‐yield graph‐theoretical analysis using standardized and not anatomical parcellations are likely Welton et al. (2015). These are further exacerbated by the unknown anatomical priors and high rate of false positives inherent to most, but especially earlier, tractography methods used (Maier‐Hein et al. 2017). Additionally, due to the unique, thin and irregular shape of the claustrum, highly accurate identification and segmentation in every subject is also paramount for reliable and specific tractography results, especially as it is in the direct vicinity of other highly connected grey matter structures (the insula and the putamen) and white matter bundles (the external and extreme capsules) (Mathur 2014).

Thus, building on these previous results, we aim to address some of the remaining research gaps by detecting human claustrum connectivity as precisely and reliably as possible using advanced techniques in both claustrum segmentation and tractography. Particularly, The issue of small sample sizes was addressed by analyzing a large human sample from the Human Connectome Project (HCP) (Glasser et al. 2013; Sotiropoulos et al. 2013), which utilized state‐of‐the‐art DWI and quality control protocols. To ensure reliable detection of claustrum connections, we reproduced our findings in a second large sample with a similar mean age and gender distribution, using imaging protocols more similar to those used in clinical routines (Wolke and Meyer 1999; Meng et al. 2016; Menegaux et al. 2021). To address the issue of false positive results and account for lacking anatomical priors, we employed a probabilistic tractography approach (Behrens et al. 2007) to analyze each tract reconstruction individually, assessing their plausibility while also providing a look at possible tract morphology. Tracking precision was further enhanced by using individual claustrum masks generated with a deep learning‐based automated claustrum segmentation tool developed in‐house (Li et al. 2021; Neubauer et al. 2022). Our overall analysis strategy included: first, analyzing claustrum connections in the HCP dataset for different connection metrics and for ipsilateral and contralateral connections to ensure robust connection detection across subjects, target regions, and metrics. Second, repeating these analyses in our second DWI dataset and comparing the results extensively with those of the HCP dataset to assess the replicability of connection detection across cohorts and different scanning protocols, independent from confound claustrum surrounding grey matter connectivity; and third, characterizing claustrum connectivity in humans in detail, given our observations of robust ipsilateral and contralateral connections.

## 2 Methods

### 2.1 Study Sample

We included in our study two age‐ and sex‐matched cohorts from previously assessed large DWI datasets, namely the Human Connectome Project, HCP, and the Bavarian Longitudinal Study (BLS). As the sample size from the BLS study was smaller than that of HCP, age‐ and sex‐matching was initiated from the BLS dataset. The HCP dataset is canonically used in the imaging community; hence, it was used as the reference cohort for primary analysis. To avoid potential confounding effects due to familial relationships, we specifically used the “100 unrelated subjects” package provided by the HCP, which ensured that no siblings or twins were included in our study.

#### 2.1.1 Participant Characteristics

##### 2.1.1.1 HCP Participant Characteristics

A total of 81 subjects were taken from the publicly available *Wu‐Minn Human Connectome Project* dataset (van Essen et al. 2013). Specifically, we used the HCP young adult *100 unrelated subjects* sub‐cohort, available for download over the online platform *ConnectomeDB* https://db.humanconnectome.org (Marcus et al. 2011). Informed consent for all subjects was obtained by HCP, and our data usage was approved by HCP and complies with all relevant ethical regulations for work with human participants. All included HCP subjects, with a gender distribution of 56% male and 44% female, were between 22 and 35 years of age (mean 28.22 ± 3.46; see Table 1 for detailed demographics).

**TABLE 1:** Demographics and scanning parameters for HCP and BLS cohort.

	HCP cohort			BLS cohort			Cohort comparison	
	(Human connectome project)			(Bavarian longitudinal study)				
	*n*	mean ± SD	Range	*n*	mean ± SD	Range	*F*(*df*)	*p*
Age (years)	81	28.22 ± 3.46	13.00	81	26.88 ± 0.74	3.30	107.17 (160)	< 0.001

	*n*	%	*n*	%	*p*
Sex (male/female)	81	56%/44%	81	64%/36%	0.336
Handedness					
Right	70	86%	63	78%	
Left	11	14%	5	6%	
Both	—	—	13	16%	

Scanning parameters		
Scanner (type)	3T	3T
Voxel size (struct. images)	0.7 × 0.7 × 0.7 mm^3^	1 × 1 × 1 mm^3^
Voxel size (diff. images)	1.25 × 1.25 × 1.25 mm^3^	2 × 2 × 2 mm^3^
*b* values	1000, 2000, and 3000 s/mm^2^	1000 s/mm^2^
Diff. weighted directions	90	32

*Note:* A two‐sample *t*‐test was used for group comparison of continuous variables (i.e., age). Fisher's exact test was used for group comparison of categorical variables (i.e., sex).

Abbreviations: BLS = Bavarian longitudinal study; Diff. = diffusion; HCP = human connectome project; SD = standard deviation; T = tesla; % = percentage of total cases.

##### 2.1.1.2 BLS Participant Characteristics

A total of 81 further non‐related healthy individuals were taken from the control arm of the Bavarian Longitudinal Study (Wolke and Meyer 1999), which investigates neonatal at‐risk children and healthy term‐born controls recruited at birth in southern Bavaria, Germany, between January 1985 and March 1986. The study and all analyses were carried out in accordance with the Declaration of Helsinki, were approved by the ethics committee of the participating university hospitals, and informed consent for all participants was obtained. For details, see (Eryigit Madzwamuse et al. 2015); briefly, *N* = 350 of the initial healthy children participating in the study were alive at 6 years, and of these, *N* = 308 were eligible for the 26‐year follow‐up, while *N* = 229 actually participated. For *N* = 102 healthy individuals, both structural T1‐weighted (T1w) and diffusion‐weighted images (DWI) were available. *N* = 21 were excluded: N = 1 had a sibling included in a study, and *N* = 20 had data quality issues (see below). *N* = 81 individuals could thus be included in the final analysis. All included BLS subjects, with a gender distribution of 64% male and 36% female, were between 25 and 28 years of age (mean 26.88 ± 0.74; see Table 1 for detailed demographics). All acquired images were checked by an experienced neuroradiologist after scanning; see Table S2 in Supporting Information for a list of incidental findings (found in *N* = 22 subjects).

#### 2.1.2 Data Acquisition

##### 2.1.2.1 HCP Data Acquisition

Imaging data acquisition for the HCP cohort was carried out at Washington University in St. Louis on the Connectome 3T MRI scanner, a customized Siemens 3T Skyra scanner with a Siemens SC 72 gradient coil and standard 32‐channel Siemens head coil, and previously described extensively (van Essen et al. 2012). In short, diffusion images were acquired with an echo‐planar imaging (EPI) sequence with the following parameters: echo time (TE) = 89.5 ms, repetition time (TR) = 5520 ms, flip angle = 78°, field of view (FOV) = 210 × 180 (RO × PE) mm^2^, matrix = 168 × 144 (RO × PE), 111 slices, voxel size 1.25 × 1.25 × 1.25 mm^3^ (isotropic); *b*‐values: 1000, 2000, and 3000 s/mm^2^; multiband acquisition (Multiband‐factor = 3). Each gradient table includes 90 diffusion weighting directions plus 6 *b* = 0 acquisitions interspersed throughout each run. Diffusion weighting consisted of three shells interspersed with an approximately equal number of acquisitions on each shell within each run. T1w (structural) images were 3D MPRAGE images acquired with the following parameters: echo time (TE) = 2.14 ms, repetition time (TR) = 2400 ms, flip angle = 8°, field of view (FOV) = 224 × 224 mm^2^, voxel size = 0.7 × 0.7 × 0.7 mm^3^ (isotropic).

##### 2.1.2.2 BLS Data Acquisition

Imaging data acquisition for the BLS cohort was carried out at two sites: the Department of Neuroradiology at the Klinikum rechts der Isar from the Technical University Munich (*N* = 59), and the Department of Radiology at the University Hospital of Bonn (*N* = 22), on 3T Philips scanners with standard 8 channel head coils and consistent sequences and parameter settings. For details, see, for example, Menegaux, Hedderich, et al. (2020) or Meng et al. (2016). Briefly, diffusion images were acquired with an echo‐planar imaging (EPI) sequence with the following parameters: echo time (TE) = 47 ms, repetition time (TR) = 20,150 ms, flip angle = 90°, field of view = 224 × 224 mm^2^, matrix = 112 × 112, 75 transverse slices, slice thickness = 2 mm, and 0 mm interslice gap, voxel size = 2 × 2 × 2 mm^3^ (isotropic); *b*‐value = 1000 s/mm^2^, 32 diffusion weighting directions. T1w (structural) images were 3D MPRAGE images acquired with the following parameters: echo time (TE) = 3.9 ms, repetition time (TR) = 7.7 ms, flip angle = 15°, field of view (FOV) = 256 × 256 mm^2^, matrix = 256 × 256, 180 sagittal slices, slice thickness = 1 mm, and 0 mm interslice gap, voxel size = 1 × 1 × 1 mm^3^. See Table 1 for an overview of the most important scanning parameters for both cohorts.

#### 2.1.3 Preprocessing of T1w‐MRIs and DWIs

##### 2.1.3.1 T1w Image Preprocessing

FSL's anatomical processing script (*fsl_anat*) was used to preprocess all T1w scans and included reorientation to standard (MNI) orientation, automatic cropping, bias‐field correction (RF/B1‐inhomogeneity‐correction), and brain extraction. The images were subsequently denoised using Pierrick Coupé's MATLAB‐based MRI Denoising Software (Coupe et al. 2008) with the Adaptive Optimized Nonlocal Means (Manjón et al. 2010) option. Finally, HCP cohort images were resampled from 0.7 to 1 mm^3^ isotropic voxel size (the same size as the BLS cohort T1w images), as the subsequently performed automated claustrum segmentation required this resolution.

##### 2.1.3.2 DWI Preprocessing

###### 2.1.3.2.1 HCP Cohort DWI Preprocessing

The HCP diffusion data were preprocessed as per the HCP diffusion preprocessing pipeline, previously described by Glasser et al. (2013) and Sotiropoulos et al. (2013). Briefly, the pipeline included intensity normalization, EPI distortion estimation using reverse phase encoding b0s with FSL *Topup* (Andersson, Skare, and Ashburner 2003), and eddy‐current and motion correction using FSL *EDDY* (Andersson and Sotiropoulos 2016).

###### 2.1.3.2.2 BLS Cohort DWI Preprocessing

Despite this data having been previously used in other studies from this lab, most recently by Hedderich et al. (2021) and Menegaux et al. (2021), we re‐preprocessed the entire sample using the newest, state‐of‐the‐art methods available. In particular, the HCP preprocessing pipeline includes distortion correction using acquired b0s with reverse phase encoding, a step previously not possible with BLS data due to such images not having been acquired. Additionally, newer versions and techniques have recently been published, that also, in some cases, allowed for the inclusion of additional subjects previously excluded due to data quality. For better comparability with the HCP data and thus more reliable overall results, we adapted our preprocessing pipeline to the steps described below.

Preprocessing of the BLS cohort started with the conversion of the acquired DICOM files to NIFTI using the dcm2nii utility, a component of the *MRIcron* tool suite (Li et al. 2016). Diffusion‐weighted images were preprocessed using the initial stages of the *PreQual* pipeline (Cai et al. 2021), which included MP‐PCA denoising, inter‐scan normalization, as well as generation of a synthetic undistorted b0 using the *SynB0‐DisCo* deep‐learning framework (Schilling et al. 2019; Schilling et al. 2020), an additional step necessary in this case as no reverse phase‐encoded images were available to use for distortion correction. FSL *Topup* (Andersson, Skare, and Ashburner 2003) was then used to perform susceptibility‐induced distortion correction using the synthetic non‐distorted image as an anatomical target (Schilling et al. 2020). After extracting the brain from the surrounding tissue using FSL *BET* (Smith 2002), FSL *EDDY* (Andersson and Sotiropoulos 2016) was used for eddy current‐induced distortion and inter‐ and intra‐volume movement correction, with the options to replace outliers (Andersson et al. 2016) and to perform slice‐wise signal dropout imputation (Andersson et al. 2017).

#### 2.1.4 Quality Control

##### 2.1.4.1 HCP Cohort Quality Control

Standardized quality check measures were implemented as part of the data acquisition and sharing process as previously described Marcus et al. (2013) and van Essen et al. (2013); briefly, images were reviewed by an HCP radiologist on overall scan quality and neuroanatomical anomalies; no individual with anomalies, clinically significant or benign, was included in the shared data. All HCP data included in the present study were additionally visually checked using the same methodology and criteria as for the BLS images prior to analysis.

##### 2.1.4.2 BLS Cohort Quality Control

The T1w (structural) and diffusion‐weighted images of each subject were carefully checked at the initial stage as well as after every subsequent phase of processing. Raw diffusion images were visually inspected and rated on a three‐point scale on the presence and strength of ghosting and chemical shift artifacts, head movement/subject motion, susceptibility‐induced distortions and noisiness as well as any other particularities that might impede or influence further analysis, such as apparent anatomical or pathological deviations. Raw structural images were equally inspected and rated on tissue contrast, spatial blurring, ringing, and other possible artifacts, especially those possibly impairing registration as well as claustrum segmentation. Based on all of these observations, each subject received an initial verdict of pass, warn, or fail. No subjects were excluded solely based on raw data quality, while 38 were rated as “warn.” Particular care was taken with these images to check whether subsequent preprocessing steps were able to sufficiently mitigate the minimal data quality issues present.

After preprocessing, group‐wise and single‐subject quantitative quality metrics were automatically derived from the data using FSL *QUAD* and *SQUAD* (Bastiani et al. 2019). Specifically, absolute motion, contrast‐to‐noise ratio (CNR), and percentage of outliers amongst all slices were analyzed. According to FSL *QUAD*‐derived measures, the included 81 BLS subjects had an average CNR of 3.51, 0.56% outliers, and an average absolute motion of 0.75 mm, which corresponds to 38% of the voxel size. Individuals with more than 2 standard deviations (SD) of deviation from the group average were automatically marked as “warn.” The diffusion tensor model fitting residuals (in the form of sum‐of‐squared‐error maps, generated through FSL *DTIFIT*) were consulted to identify significant artifact impact. Corrected data was also visually assessed on the impact of present artifacts on tensor fitting and fractional anisotropy (FA) measures, non‐corrected excess motion and noise, anatomical consistency of tensor fit, and overall quality. All subjects marked as “warn” during one of the previous steps underwent additional visual inspection and consultation with other researchers experienced in DWI quality checks to ensure that preprocessing adequately remedied the issues and that the resulting data was suitable for further analysis. Based on all observations, each subject finally received a final verdict of pass or fail. A verdict of fail led to the exclusion of that particular subject from all further analysis. As noted above, a failed quality check led to the exclusion of a total of *N* = 20 individuals: *N* = 14 due to ghosting artifacts, *N* = 4 due to both ghosting artifacts and excessive noise, *N* = 1 due to both ghosting artifacts and excessive motion leading to signal loss, and *N* = 1 due to a missing slice.

### 2.2 Claustrum Segmentation

The bilateral claustra were individually delineated in each subject's T1w image using a multi‐view deep learning‐based automated claustrum segmentation tool developed in‐house (Hedderich et al. 2021; Li et al. 2021). Each individual mask was checked for maximum accuracy in each slice and manually adjusted in ITK‐SNAP 3.6.0 (Yushkevich et al. 2006) when needed by J.W. The standardized segmentation protocol for manual annotation followed for checking the delineations was previously described in detail by Li et al. (2021) and applied in the BLS cohort by Hedderich et al. (2021).

### 2.3 Claustrum Tractography

#### 2.3.1 Registration

Advanced normalization tools package (Avants et al. 2008; Avants et al. 2011) was used to register the images in different spaces to each other and obtain standardized MNI space representations of all subsequent tract reconstructions initially obtained in diffusion space. Each subject's brain‐extracted b0‐image was registered to the brain‐extracted structural T1w image using the linear algorithm. Then, the structural T1w images were registered to standard (MNI‐152) space (Brett, Johnsrude, and Owen 2002) using the ANTs diffeomorphic (non‐linear) registration algorithm (*antsRegistrationSyN*), available on GitHub (https://github.com/ANTsX/ANTs). This resulted in transformation matrices between diffusion and structural space as well as a non‐linear warp field between structural space and standard MNI space, allowing for the transformation of images as well as masks and tractography outputs into each space as needed. Additionally, diffusion images transformed into structural space and structural T1w images transformed into standard MNI space were generated and visually checked to assess the quality of the transformation.

#### 2.3.2 Cortical and Subcortical Target ROI Creation

**FIGURE 1:**
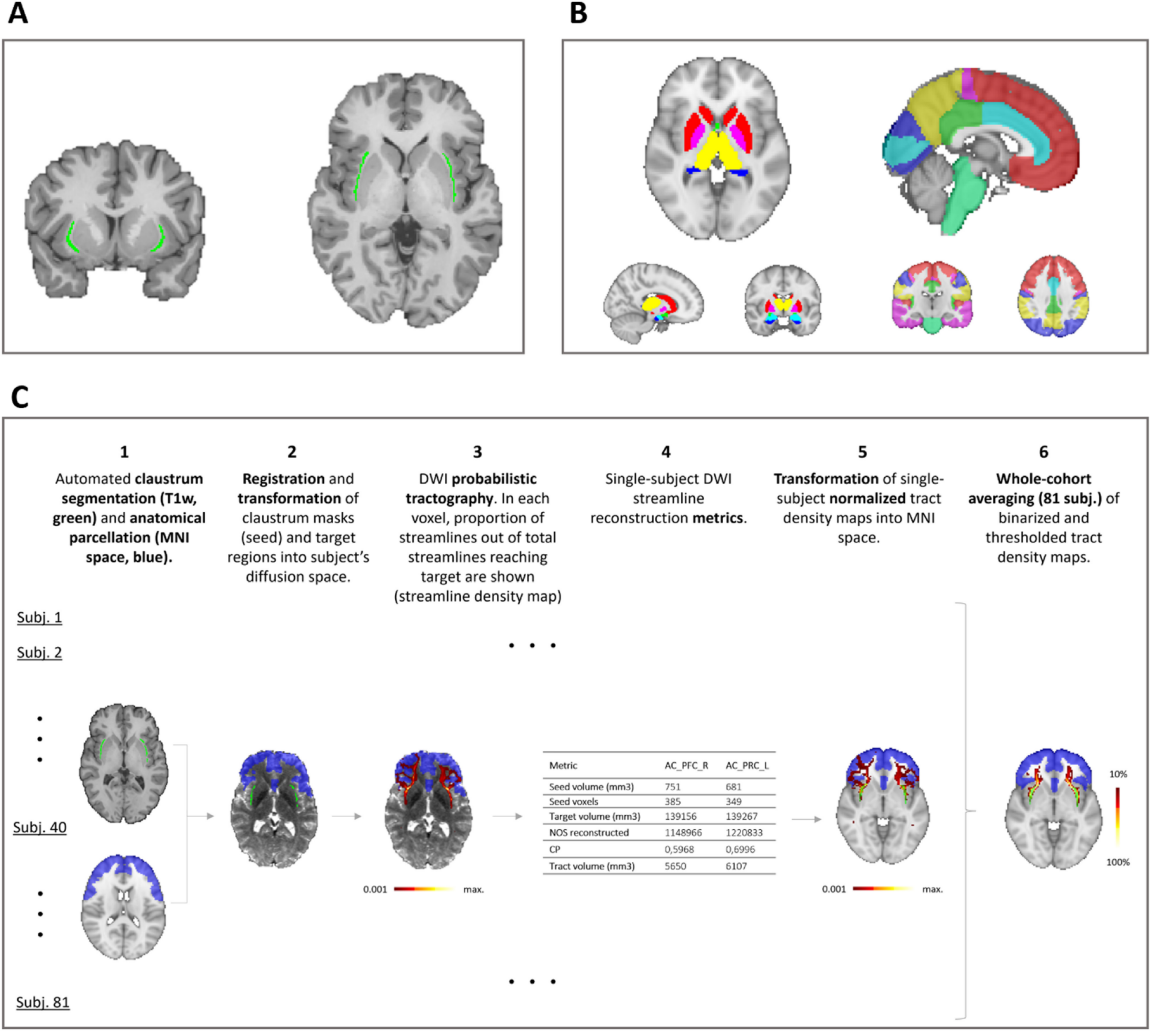
Investigating claustrum connectivity using DWI‐based tractography—methods overview. (A) The claustrum, seed region for tractography: The bilateral claustra, segmented using an automated segmentation algorithm, are shown here in green on a coronal and an axial slice of one exemplary subject's skull‐stripped T1w structural image. (B) Tractography targets. 13 cortical and 9 subcortical ROIs were defined as targets for claustrum connectivity reconstruction using probabilistic tractography, shown here in MNI‐space on the MNI152 standard‐space T1w average structural template brain. (C) Workflow of tractography with outcomes, shown for right and left prefrontal cortices. AC_PFC_L = left prefrontal cortex; AC_PFC_R = right prefrontal cortex; CP = connection probability; DWI = diffusion tensor imaging; MNI = Montreal Neurological Institute; NOS = number of streamlines; subj. = subject; T1w = T1‐weighted.

Based on previous findings of regions connected to the claustrum in non‐human mammals or humans as well as functional considerations (Torgerson et al. 2015; Brown et al. 2017; Wang et al. 2017; Jackson, Smith, and Lee 2020), we defined 13 cortical and 9 subcortical target ROIs (see Figure 1B and Table 2), for which binary masks were created in standard space. Cortical targets were subdivided into primary cortices, associative cortices and limbic cortices. For primary cortices, primary motor, somatosensory, visual, and auditory cortex masks were defined using the intersection between the widely‐used Harvard‐Oxford cortical atlas (Desikan et al. 2006) and Juelich microstructural atlas (Amunts et al. 2020), the latter providing added resolution to the former's mainly macroanatomical definition through microstructural information. For associative and limbic cortices, masks for prefrontal, parietal, occipital, and temporal associative cortices as well as anterior and posterior insular cortices, anterior and posterior cingulate cortices, and hippocampi were defined using the Harvard‐Oxford cortical atlas (Desikan et al. 2006). As this atlas only defines the insula as a whole, a 3D probabilistic atlas of the insula (Faillenot et al. 2017) was used to make the subdivision into the anterior and posterior insula.

**TABLE 2:** Target ROIs and source atlases.

	Target	Abbreviation	Left/Right	Atlas based on
Cortical targets				
Primary cortices	Primary motor cortex	*PC_mot*	Yes	Juelich microstructural atlas and Harvard‐Oxford cortical atlas
	Primary somatosensory cortex	*PC_sens*	Yes	
	Primary visual cortex	*PC_vis*	Yes	
	Primary auditory cortex	*PC_aud*	Yes	
Associative and limbic cortices	Prefrontal associative cortex	*AC_PFC*	Yes	Harvard‐Oxford cortical atlas, Faillenot et al. (2017)
	Parietal associative cortex	*AC_Par*	Yes	
	Occipital associative cortex	*AC_Occ*	Yes	
	Temporal associative cortex	*AC_Temp*	Yes	
	Anterior insular cortex	*Ins_ant*	Yes	
	Posterior insular cortex	*Ins_post*	Yes	
	Anterior cingulate cortex	*C_ant*	Yes	
	Posterior cingulate cortex	*C_pos*	Yes	
	Hippocampus	*SC_Hip*	Yes	
Subcortical targets				
Basal ganglia	Striatum	*SC_BG_Str*	Yes	Harvard‐Oxford subcortical
	Pallidum	*SC_BG_Pal*	Yes	
	Subthalamic nucleus	*SC_BG_SN*	Yes	
Neuromodulatory nuclei	Raphe nuclei	*SC_NM_R*	No	AAN
	Dopaminergic neuromodulatory nuclei	*SC_NM_Dop*	No	AAN, Pauli, Nili, and Tyszka (2018)
	Cholinergic basal forebrain	*SC_NM_cBF*	No	Fritz et al. (2019)
	Locus coeruleus	*SC_NM_LC*	Yes	AAN
Other subcortical	Amygdala	*SC_Amy*	Yes	Harvard‐Oxford subcortical atlas
	Thalamus	*SC_Thal*	Yes	

*Note:* See Table S1 in Supporting Information for more details on the exact subregions used for the creation of each mask.

Abbreviations: AAN = ascending arousal network; ROI = region of interest.

For subcortical targets, the basal ganglia (striatum, pallidum, and subthalamic nucleus) as well as the amygdala and thalamus were delineated using the Harvard‐Oxford subcortical atlas, obtained from NeuroVault (Gorgolewski et al. 2015). For the neuromodulatory nuclei, masks for the raphe nuclei and locus coeruleus were obtained from the Harvard Ascending Arousal Network (AAN) atlas (Edlow et al. 2012). The dopaminergic neuromodulatory nuclei mask was made by combining the AAN's ventral tegmental area (VTA) mask with Pauli and colleagues' substantia nigra compacta (SNc) mask (Pauli, Nili, and Tyszka 2018). Finally, Fritz and colleagues' cholinergic basal forebrain mask (Fritz et al. 2019) was used to complete the subcortical ROIs.

The previously described transformation matrices and warp‐fields were (inversely) applied to all these ROI masks defined in standard MNI space in order to align them into each subject's diffusion space through nearest neighbor interpolation. The resulting individual masks were once more visually checked to ensure a good fit.

#### 2.3.3 Probabilistic Tractography

FSL 6.0.1 bedpostX (Bayesian Estimation of Diffusion Parameters Obtained using Sampling Techniques, FSL) runs Markov Chain Monte Carlo sampling and was used to calculate the distribution of likely fiber orientations in each voxel (Behrens, Woolrich, et al. 2003; Behrens et al. 2007). For the HCP cohort, bedpostX output data, obtained using the HCP bedpostX pipeline, was available for download in the diffusion analysis package and was used with no modification. Here, fiber orientations were modelled using the available multi‐shell information from all three *b*‐values (1000, 2000, and 3000 s/mm^2^) (Sotiropoulos et al. 2013). For the BLS cohort, as only one non‐zero *b*‐value was acquired (1000 s/mm^2^), the single‐shell model was used, and two fibers per voxel were modelled. All other options were left as default.

FSL 6.0.1 *probtrackx2* (Behrens, Woolrich, et al. 2003; Behrens, Johansen‐Berg, et al. 2003; Behrens et al. 2007) was then used to generate streamlines between the defined seed and target regions, resulting in a histogram of how many streamlines connecting the two regions visited each voxel, as well as the number of streamlines connecting the regions. The default settings were used for tracking: curvature threshold of 80°, 5000 streamlines per voxel, and 0.5 mm step length. In order to accelerate this step, the GPU version of probtrackx2 was used (Hernandez‐Fernandez et al. 2019). The individual claustrum masks, transformed into each subject's diffusion space, were thus used as seeds to generate streamline reconstructions to each of the ROIs described above. The target ROI was defined as termination AND waypoint mask, meaning only those streamlines that reached the target were retained, and the streamline was terminated as soon as it would exit the target ROI. Tracking was conducted separately for each hemisphere, ipsilaterally as well as contralaterally. For the generation of ipsilateral streamlines, a mask of the contralateral hemisphere was used as an exclusion mask to terminate any streamlines crossing into the opposite hemisphere.

This resulted in a reconstruction showing, in each voxel, the number of streamlines meeting the defined inclusion and exclusion criteria and passing through that voxel (probabilistic visitation map), as well as in a number, the so‐called waytotal, representing the total number of streamlines, out of all of those seeded, that reached the target ROI (Figure 1C). As there were 19 bilateral target ROIs and 3 unilateral target ROIs (see Table 2), a total of 82 streamline reconstructions were performed per subject.

### 2.4 Connectivity Metrics and Statistical Analysis

#### 2.4.1 Streamline Reconstruction Analysis

For inter‐subject comparability, the individual probabilistic visitation maps were normalized by dividing each by the waytotal, resulting in a map showing, in each voxel, the portion of streamlines reaching the target ROI passing through that voxel (Berndt et al. 2019; Menegaux et al. 2019; Menegaux et al. 2021). Each subject's streamline tract (referring to the “tract” formed by the total of reconstructed streamlines originating in the claustrum and reaching the target ROI) was then transformed to standard MNI space using the transformation matrices from the ANTs registration procedure described above (Figure 1C). After thresholding individual maps at 1% to reduce false negatives (Warrington et al. 2020), the variance map of the individual subject's streamline tracts, then the mean of all streamline tracts, was calculated using fslmaths (Menegaux, Bäuerlein, et al. 2020; Menegaux et al. 2021). This resulted in a cohort average map in MNI space showing, in each voxel, what proportion of subjects had their streamline tract passing through that voxel.

#### 2.4.2 Connectivity Metrics

There is not a lot of consensus in the available literature on the best way to quantify white matter connectivity as examined by probabilistic tractography. Streamline count, or waytotal, is a highly variable measure in itself and very dependent on the number of seeded streamlines (directly relating to seed volume), amongst other things (Bajammal, Yoldemir, and Abugharbieh 2015). However, two methods representing different aspects of streamline connectivity are regularly used and were employed in our study to further describe the streamline reconstructions.

First, connection density (CD) is calculated as the number of streamlines reaching the target region (NOS, Waytotal) divided by the total volume of connected grey matter regions and representing the density of streamlines connecting the two regions by metric volume (Cheng et al. 2012; van den Heuvel et al. 2015; Gu et al. 2021).

Second, connection probability (CP): number of streamlines reaching the target region divided by the total number of streamlines seeded from the seed region (num. of seeded streamlines per voxel × num. of source points in the seed region), thus representing the probability that a seeded streamline actually reaches the target region (Yo et al. 2009; Cao et al. 2013; Tsai 2018).

$$\mathrm{CD}=\frac{\mathrm{NOS}}{\mathrm{See}d_{\mathrm{vol}}+\text{Targe}t_{\mathrm{vol}.}}$$

$$\mathrm{CP}=\frac{\mathrm{NOS}}{5000\times \mathrm{See}d_{\mathrm{vox}}}$$

We applied these metrics to all streamline reconstructions made in order to obtain, across our two cohorts, a complete picture, both qualitatively and quantitatively, of streamline connectivity of the claustrum as shown by probabilistic diffusion tractography.

#### 2.4.3 Statistical Analysis

SPSS statistics package version 28 (IBM) was used for all statistical analyses. All values reported in the format “*x* ± *y*” throughout the manuscript represent the mean ± SD unless otherwise stated.

For descriptive statistics comparing the two cohorts, a two‐sample *t*‐test was used for the group comparison of continuous variables (e.g., age), and Fisher's exact test was used for categorical variables (e.g., sex). To directly compare connectivity metrics between cohorts, average CD and CP metrics across all target regions from the HCP and BLS cohorts were correlated using Pearson's correlation. After ranking all target regions by their average CD and CP values in descending order for each cohort, CD and CP rankings were also correlated using Spearman correlation. Additionally, we assessed the linear relationship between CD and CP variables of all right‐side ipsilateral tractography results using Pearson's correlation.

For the control analysis examining potential contamination of claustrum connectivity from surrounding brain matter connectivity, we performed the following statistical tests: first, to ensure that there is significant claustrum connectivity towards a given target region, a one‐sample *t*‐test was conducted for corresponding connectivity metrics. Next, a linear regression model was fitted to test the independence of claustrum connectivity metrics from the connectivity metrics of the directly neighboring regions, namely the insula and the putamen. The tractography for this control analysis was conducted with the same settings and methodology as described for the claustrum, with the only difference being the use of insula and putamen masks as seed regions.

To compare the spatial distribution of tracts between the HCP and BLS datasets, we used FSL's “fslcc” function, which performs voxel‐wise correlation between 3D images. This method, previously used by Warrington and colleagues in their study on XTRACT, an automated tractography protocol implemented in FSL (Warrington et al. 2020), provided a robust approach to comparing probabilistic tractography between subjects and cohorts. We used this function in two ways: first, to correlate the cohort‐average tract maps thresholded at 10% (0.1) and second, to correlate individual subject tractography results both within and across cohorts. Correlations were run for each possible subject pair (i.e., for BLS and HCP comparison, for a total of 81 × 81 = 6561 subject pairs), and mean correlation values across all these individual pair correlations were reported for each target region.

We also used dice coefficient (DC) to quantify the spatial overlap between cohort‐average tract maps of the HCP and BLS datasets, again thresholded at 10%, defined as follows:

$$\mathrm{DS}=\frac{2\left(\mathrm{HCP}\cap \mathrm{BLS}\right)}{\left|\mathrm{HCP}\right|+|\mathrm{BLS}|}$$

## 3 Results

### 3.1 Sample Characteristics

All relevant sample characteristics are summarized in Table 1. While the reference (HCP) and control (BLS) cohorts did not differ significantly for sex (*p* = 0.336), the HCP cohort had significantly (*p* < 0.001) higher mean age (28.22 ± 3.46 years) than the BLS cohort (26.88 ± 0.74 years), as well as a considerably larger range (13.00 years for HCP, 3.30 years for BLS). This difference needs to be taken into account when examining differing results, as white matter connectivity is significantly influenced by age and changes during development (Lebel et al. 2012).

### 3.2 Robust Detection of Claustrum Connections via DWI‐Based Tractography

#### 3.2.1 Consistent Tractography Across Subjects and Regions Within the Ipsilateral Hemisphere

To examine consistent streamline connectivity of the claustrum across subjects and target regions, we performed probabilistic tractography from the claustrum to all ipsilateral target ROIs for each subject of the HCP data set (shown in Figure 1B and Table 2). The individual steps, from tractography in each subject's diffusion space to obtaining whole‐cohort averaged streamline connectivity maps, are shown in Figure 1.

**FIGURE 2:**
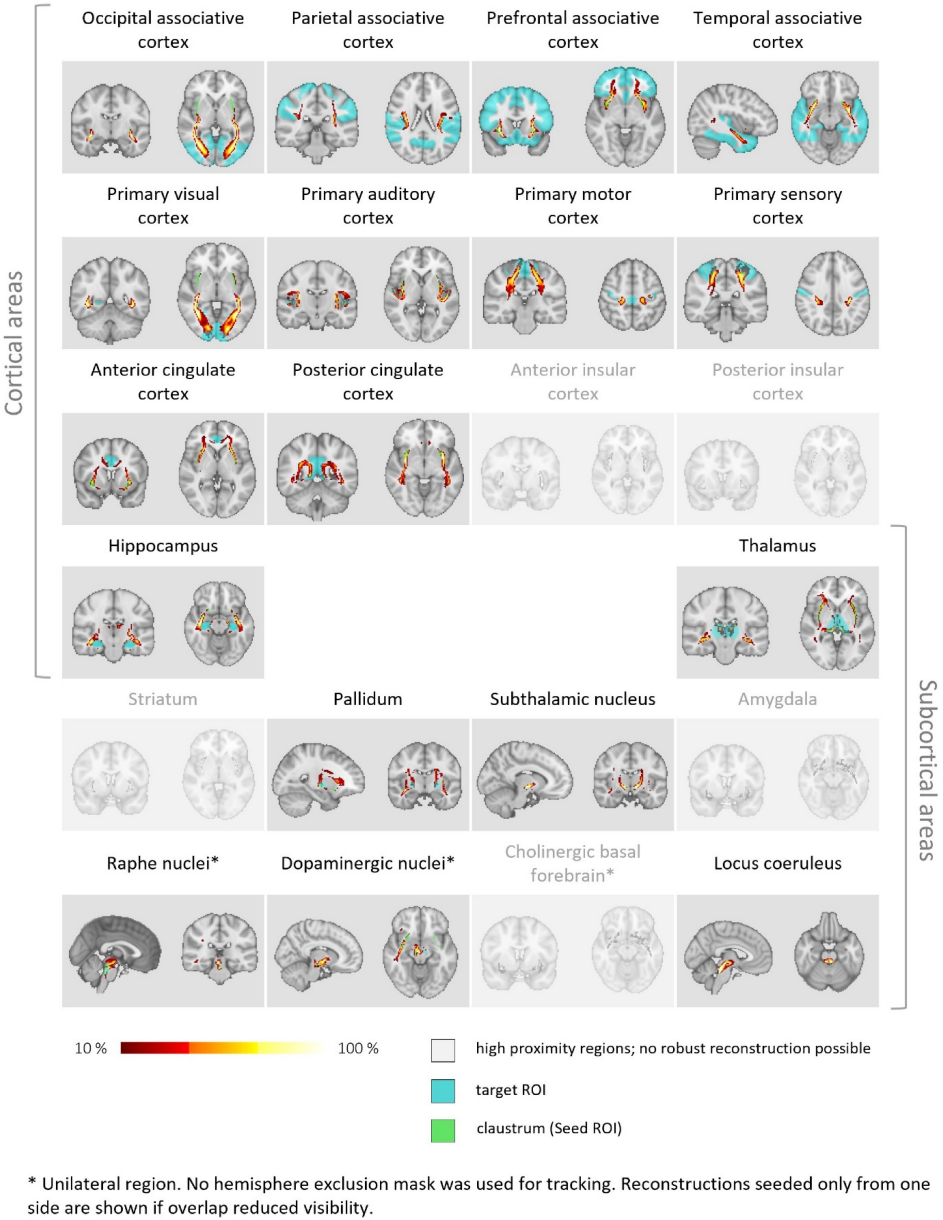
Ipsilateral claustrum connectivity: Ipsilateral streamlines across HCP cohort individuals. The cohort‐averaged reconstructed streamline tracts, depicting in each voxel the percentage of subjects having their individual streamline tract passing through that voxel, are displayed overlaid on the MNI‐152 T1 2mm brain. Ipsilateral reconstructions in both hemispheres are shown. The representation is thresholded at 10% (lower threshold). HCP = human connectome project; ROI = region of interest.

In order to ensure the feasibility of streamline connectivity reconstruction to all chosen target ROIs, we carefully examined the resulting streamlines in all individual subjects before averaging them across the cohort to identify potential anatomical inconsistencies or other issues. We found reliable streamline detection across subjects for almost all target regions except those nearby the claustrum. In more detail, the main complicating factor for streamline detection we identified was anatomical proximity. Certain regions of interest are directly adjacent or separated from the claustrum by a few voxels only, such that streamline reconstruction by probabilistic tractography was not assessable, and any regions directly adjacent to the claustrum or only separated by 1–2 voxels in MNI space were consequently excluded. This proximity alone can yield a significant number of false‐positive streamlines, undermining the reliability of the detected pathways. Additionally, these regions might have overlapping voxels, rendering tractography and the analysis of any corresponding connectivity metrics meaningless. Due to these considerations, we excluded 5 target ROIs: anterior and posterior insula, cholinergic basal forebrain, amygdala, and striatum (Figure 2, Table 2). We conclude that pathways reconstructed using FSL probtrackx pertaining to these regions are not reliable. Special care should be taken to exclude these regions to avoid inaccuracies, especially in automated or high‐yield connectivity analyses.

Besides this, we found that, after across‐cohort averaging, robust streamline reconstruction to all other areas was possible, anatomically plausible, and resulted in tract‐like morphology. A slice of the resulting streamline tract, depicting in each voxel the percentage of subjects having their individual streamline tract passing through that voxel, is shown in Figure 2. The representation is thresholded at 10%. This resulting morphology is remarkable, especially considering the typically high between‐subject variability of white matter tracts (Bürgel et al. 2006; Thiebaut de Schotten et al. 2011). Visually, we conclude that consistent streamline reconstruction across subjects is possible for all ipsilateral regions besides the noted exceptions; this provides further evidence for widespread ipsilateral connectivity of the claustrum.

**FIGURE 3:**
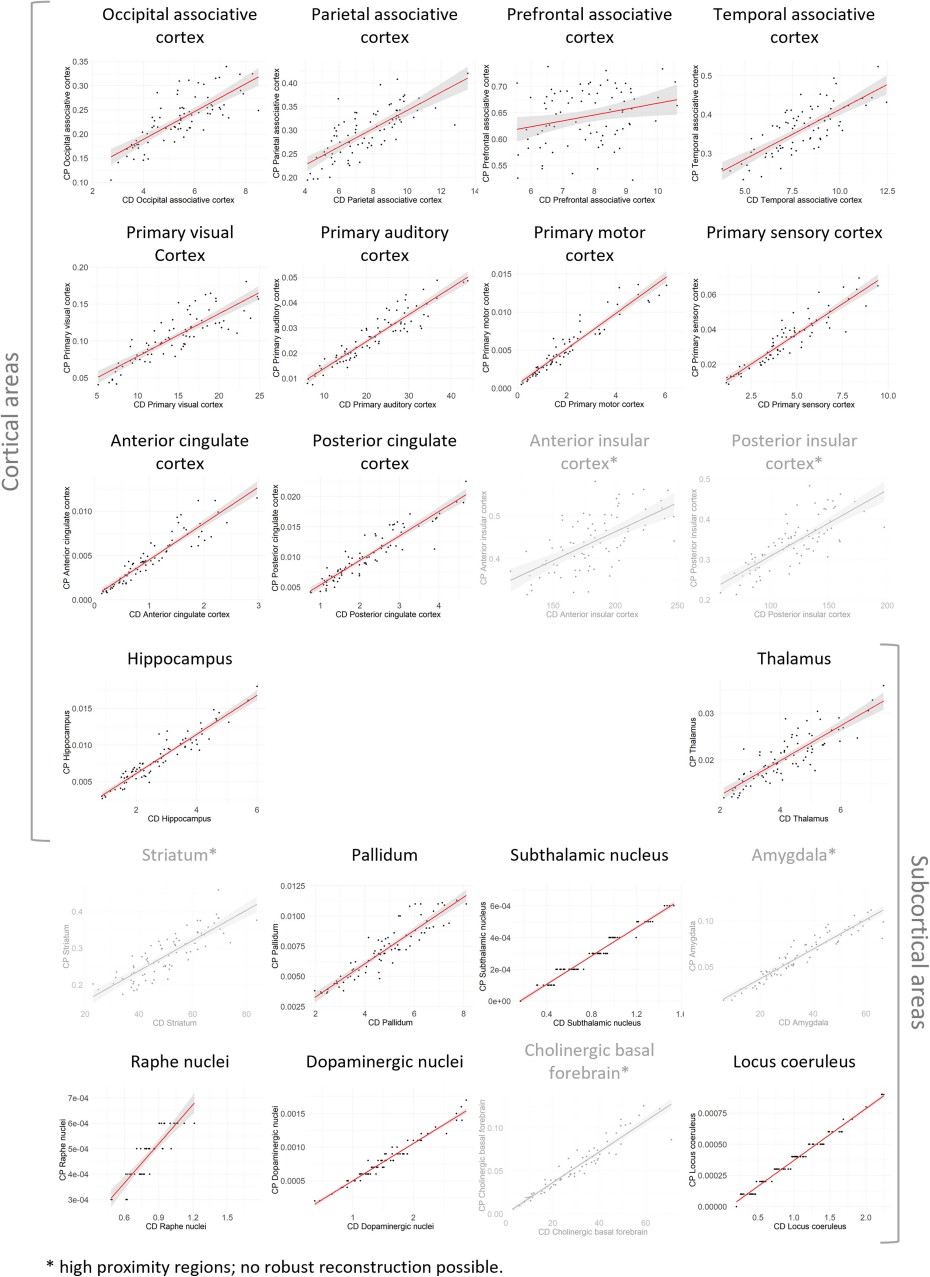
Ipsilateral claustrum connectivity: Comparing outcome measures. Pearson's correlations between CD and CP derived for all examined ROIs show highly significant (< 0.001) associations, except for the prefrontal cortex (*p* = 0.019). Scatterplots with the corresponding regression line are shown here for ipsilateral right side hemisphere connectivity metrics in the HCP cohort. See Table S5 in Supporting Information for measures and Pearson correlation coefficients for every region. CD = connection density; CP = connection probability; HCP = human connectome project; ROI = region of interest.

Finally, to further examine the consistency of the detection of streamline connections across regions, we investigated whether the two different measures we used to quantify claustrum connectivity, correlate for different regional targets in largely the same way. To this end, we correlated CP and CD for each target region across subjects. Pearson's correlation between CD and CP measures derived for all examined target regions show highly significant (< 0.001) correlations; only for the prefrontal associative cortex, the significance was *p* = 0.019 (Figure 3, Table S5). This result demonstrates that streamline reconstruction is largely consistent for different connection outcomes across the different cortical and subcortical targets.

#### 3.2.2 Replicable Tractography Across Scanners and Sequences

To ensure that our findings were comparable across different cohorts with different acquisition parameters, we reproduced all findings of ipsilateral connections in a second, independent cohort of healthy adults, the BLS cohort. As is summarized in Table 1, the most significant differences in scanning parameters between the HCP and BLS cohorts are—beyond different scanner types—voxel size of the diffusion‐weighted images, the number of diffusion‐weighted directions, and, most importantly, the number of shells. For voxel size, HCP had a higher resolution at 1.25 mm^3^ isotropic when compared to 2 mm^3^ isotropic for BLS. The HCP cohort was also acquired with multiple shells (3 distinct *b*‐values) and 90 diffusion‐weighted directions when compared to a single shell and 32 diffusion‐weighted directions for BLS. As such, the BLS cohort was acquired with not state‐of‐the‐art, but more widely clinically available and easily implemented parameters.

**FIGURE 4:**
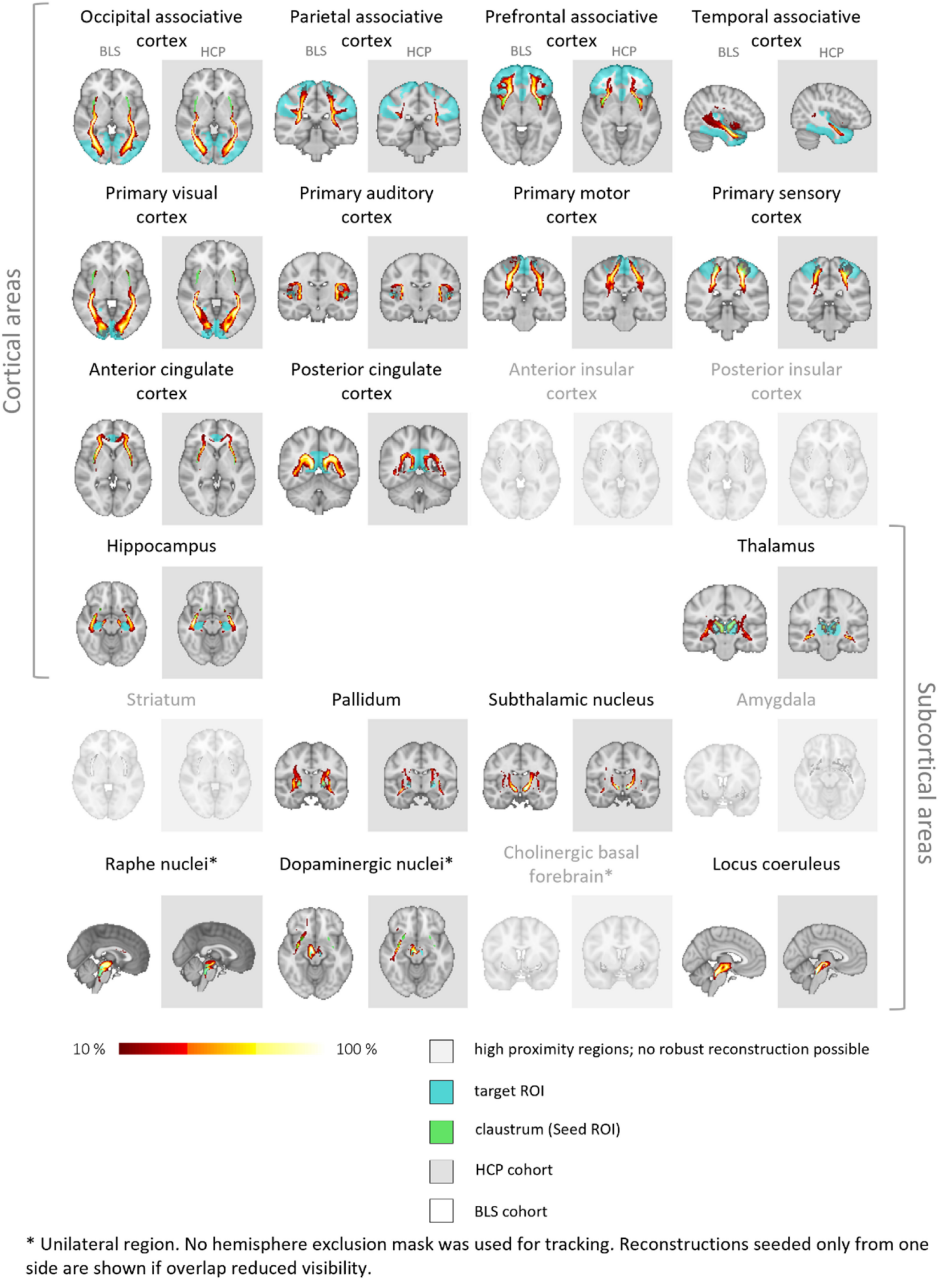
Ipsilateral claustrum connectivity: Ipsilateral streamlines, comparison between cohorts. The cohort‐averaged reconstructed streamline tracts, depicting in each voxel the percentage of subjects having their individual streamline tract passing through that voxel, are displayed overlaid on the MNI‐152 T1 2mm brain. The representation is thresholded at 10% (0.1; lower threshold). BLS = Bavarian longitudinal study; HCP = human connectome project; ROI = region of interest.

Figure 4 shows side‐by‐side comparisons of streamline reconstructions to each target ROI in the BLS and HCP cohort, always in the same slice. Despite diverging scanning parameters, a reproduction of the streamline tracts found in the HCP cohort was possible, and morphologically highly similar, judging by visual inspection, in the BLS cohort, throughout all target ROIs determined to be of sufficient distance to the claustrum as detailed previously. The BLS cohort's tract reconstruction had a slightly stronger dispersion of streamline tracts; this may be attributable to the fact that by using only single‐shell information, the tracking was less precise, especially in voxels with crossing fibers.

**FIGURE 5:**
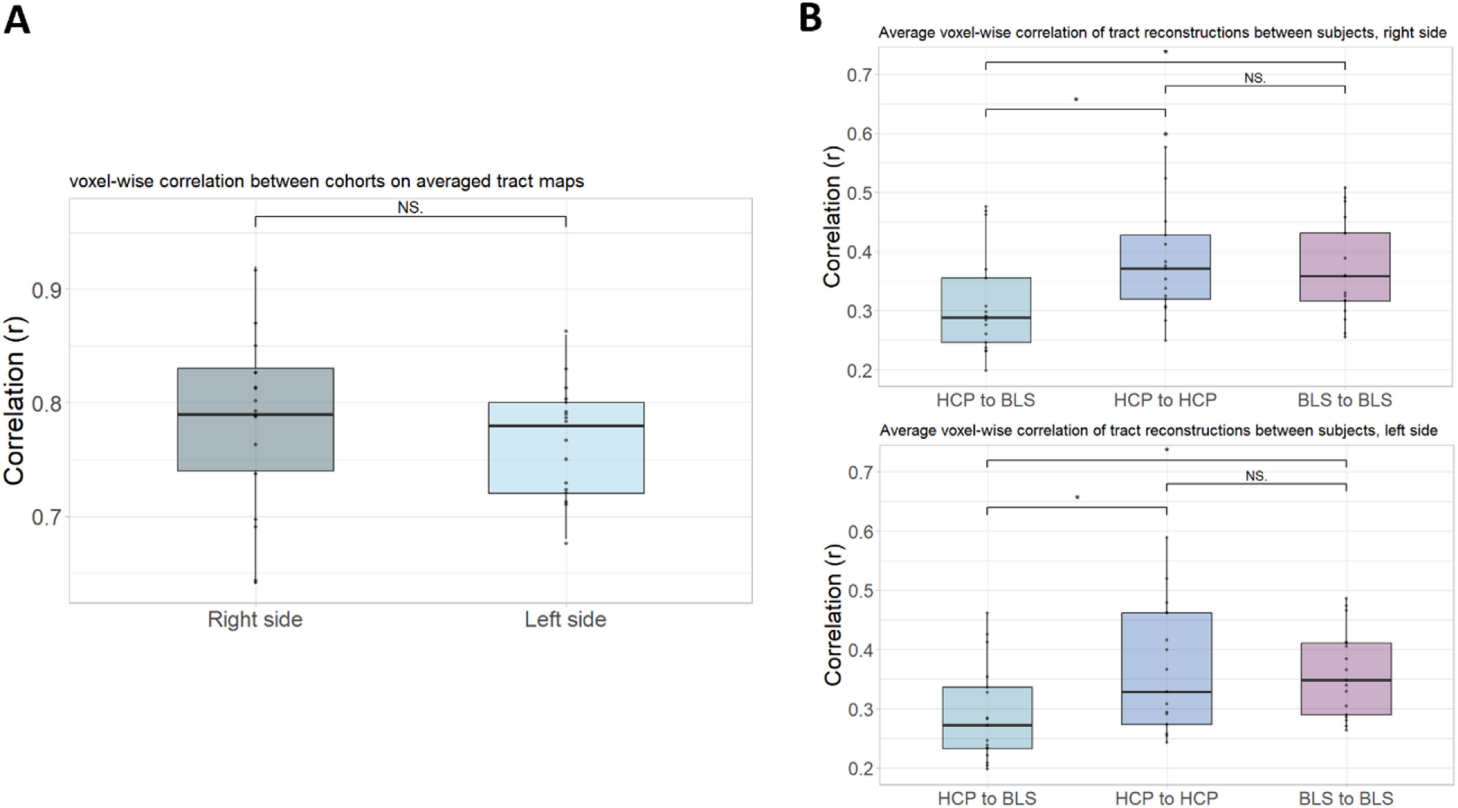
Summary of inter‐cohort robustness. (A) Boxplots of the voxel‐wise correlation between HCP and BLS cohorts on cohort‐averaged tract maps thresholded at 10% (0.1) across all reconstructed tracts. (B) Boxplots of the average correlations between 6561 subject pairs within and across cohorts across all reconstructed tracts. Individual subject's streamline reconstructions were thresholded at 1% (0.01; lower threshold) and normalized to MNI space. Plots are shown for right side (above) and left side tractography (below); see Supporting Information for all values (Tables S7 and S8). BLS = Bavarian longitudinal study; HCP = human connectome project; NS = not significant; * = statistically significant difference at 5% level (*p* < 0.05).

To compare tract reconstructions between the two cohorts and test the reliability of claustrum connectivity across datasets, both at the cohort average and at the individual subject level, we performed voxel‐wise correlations using FSL fslcc (Figure 5, and Tables S7 and S8). When comparing cohort‐average tract maps, we found a high correlation between HCP and BLS cohorts (Ø 0.77 ± 0.05 and 0.78 ± 0.08 for left and right sides, respectively). For correlation between all individual subjects, across‐cohort comparison gave an average correlation of 0.29 ± 0.08 for the left and 0.31 ± 0.09 for the right sides. This subject‐subject correlation was lower than the within‐cohort comparisons, something to be expected due to the differences in data quality and scanning parameters: within the HCP cohort, there was an average correlation of 0.37 ± 0.11 and 0.39 ± 0.10 for left and right sides, and within the BLS cohort, an average correlation of 0.36 ± 0.07 and 0.37 ± 0.08 for left and right sides. These numbers are roughly in line with values found by Warrington and colleagues in a similar analysis of large white matter tracts in the human brain, although not specific to a singular seed region (Warrington et al. 2020). These two analyses indicate a generally consistent spatial distribution of tracts between the HCP and BLS cohorts, supporting the robustness of our findings.

In addition to voxel‐wise correlations, we calculated the Dice coefficient (DC) to assess volumetric overlap between HCP and BLS cohort average tract maps, thresholded at 10% (Table 3). Detailed tract volumes and overlap percentages for both cohorts are provided in Table S6 in the Supporting Information. DC values indicated moderate agreement for both ipsilateral and contralateral connections, with values of Ø 0.59 ± 0.07 and 0.61 ± 0.09 for the left and right ipsilateral sides, and Ø 0.56 ± 0.09 for the left and 0.54 ± 0.12 for the right contralateral sides. This level of overlap is consistent with what is regarded as moderate agreement in the literature (Wilson et al. 2017; Kreilkamp et al. 2019). When looking at the distinct target ROIs, among the ipsilateral regions, the highest volumetric overlap was observed in the primary somatosensory cortex (0.678 for the left and 0.723 for the right) and the primary visual cortex (0.674 for the left and 0.734 for the right). The lowest overlap was found in the associative parietal cortex (0.507 for the left and 0.437 for the right) and the right hippocampus (0.456). Overall, these findings indicate a generally moderate volumetric overlap between the HCP and BLS cohorts.

**TABLE 3:** Cohort‐average ipsilateral tract atlas comparison between datasets using dice coefficient (DC).

Target ROI	DC left side	DC right side
AC_Occ	0.570	0.542
AC_Par	0.507	0.437
AC_PFC	0.544	0.524
AC_Temp	0.575	0.552
C_ant	0.650	0.660
C_pos	0.672	0.702
PC_aud	0.691	0.686
PC_mot	0.660	0.714
PC_sens	0.678	0.723
PC_vis	0.674	0.734
SC_BG_Pal	0.538	0.562
SC_BG_SN	0.617	0.640
SC_Hip	0.564	0.456
SC_NM_Dop	0.541	0.568
SC_NM_LC	0.581	0.650
SC_NM_R	0.466	0.560
SC_Thal	0.577	0.601
Mean**s**	**0.594 ± 0.067**	**0.607 ± 0.091**

*Note:* DC was calculated for each target region between corresponding tracts in HCP and BLS cohorts using cohort‐averaged tracts thresholded at 10% (0.1). Please refer to Table S9 in Supporting Information for data on contralateral connectivity.

Abbreviations: BLS = Bavarian longitudinal study; DC = dice coefficient; HCP = human connectome project; ROI = region of interest.

We conclude that, for the designated regions, probabilistic tractography is a reliable method of reconstructing and examining claustrum connectivity, and that it is robust against sample and method of acquisition.

**FIGURE 6:**
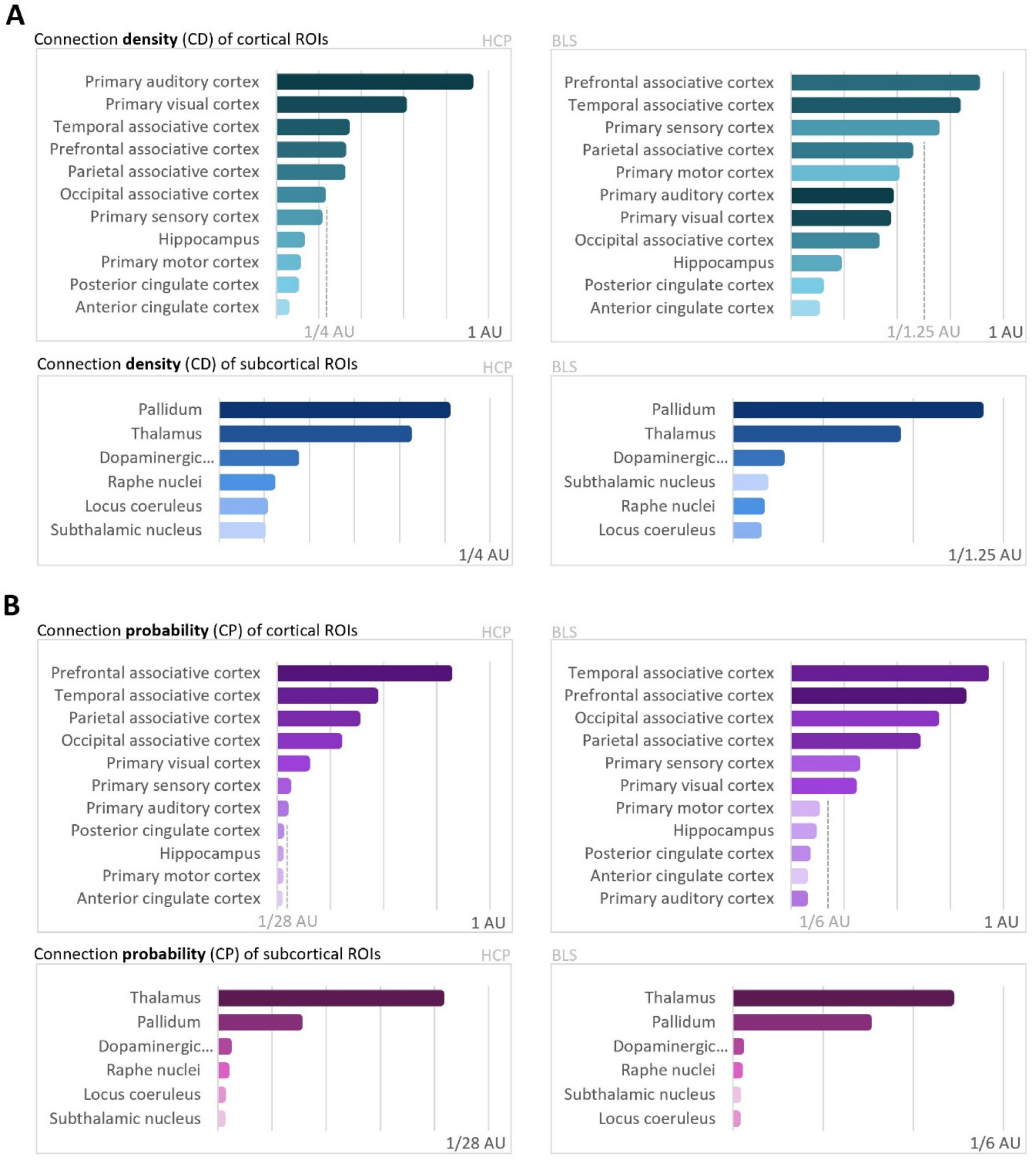
Ipsilateral claustrum connectivity: Outcomes across cohorts. (A) Connection density of cortical and subcortical ROIs for HCP (left) and BLS (right) cohorts. (B) Connection probability of cortical and subcortical ROIs for HCP (left) and BLS (right) cohorts. Metrics are shown for cortical and subcortical areas separately, and for the HCP cohort (left side) as well as the BLS cohort (right side). As connectivity metrics are highly influenced by scanning parameters and image characteristics, comparing absolute values between different datasets is difficult. Rather, the order of regions sorted by CD and CP values, as is displayed here, can serve to show which regions are relatively more or less connected to the claustrum. For this reason, only arbitrary units are indicated. The order of regions differs most for CD of cortical ROIs (A, upper panels), although the least connected regions are still preserved across the two cohorts. For CP of cortical ROIs (B, upper panels), the relative order of regions is highly similar, and for both CD and CP of subcortical ROIs, nearly identical between HCP and BLS cohorts. AU = arbitrary unit; BLS = Bavarian longitudinal study; CD = connection density; CP = connection probability; HCP = human connectome project; ROI = region of interest.

Finally, we visualized the pattern of relative CD and CP across regions to compare the connectivity patterns revealed by the strength of streamline reconstruction between the two cohorts (Figure 6). For CD (Figure 6A), a more significant difference was seen for the cortical ROIs, mainly because the primary auditory and primary visual cortices did not have as high of a CD when compared to the associative cortices in the BLS cohort as was the case in the HCP cohort. For subcortical ROIs, the pattern of CD values was very closely matched in both cohorts. For CP (Figure 6B), the pattern was nearly identical for cortical and subcortical regions. We also correlated both the CD and CP metrics between datasets (Pearson's correlation) as well as their rankings (Spearman correlation) to quantitatively compare the similarity of these metrics across the two cohorts (Table 4). When correlating CD and CP metrics between the two cohorts, we observed a moderate, statistically significant correlation for CD on the left side (*r* = 0.635, *p* = 0.006), while the right side only showed a trend towards significance (*r* = 0.466, *p* = 0.059). In contrast, CP demonstrated a strong and highly significant correlation for both sides (*r* = 0.980, *p* < 0.001 for left and *r* = 0.981, *p* < 0.001 for right). Spearman correlation of the resulting rankings between the BLS and HCP cohort showed a strong and highly significant correlation for both CD (*r* = 0.821, *p* < 0.001 and *r* = 0.779, *p* < 0.001 for L and R sides) and CP (*r* = 0.958, *p* < 0.001 and *r* = 0.934, *p* < 0.001 for L and R sides).

**TABLE 4:** Correlation of CD and CP metrics between datasets.

	Pearson's correlation of metrics between datasets		Spearman correlation of metric rankings between datasets	
	Left side	Right side	Left side	Right side
CD	*r*(15) = 0.635, *p* = 0.006	*r*(15) = 0.466, *p* = 0.059	*r*(15) = 0.821, *p* < 0.001	*r*(15) = 0.779, *p* < 0.001
CP	*r*(15) = 0.980, *p* < 0.001	*r*(15) = 0.981, *p* < 0.001	*r*(15) = 0.958, *p* < 0.001	*r*(15) = 0.934, *p* < 0.001

*Note:* Pearson's correlation was used to calculate the correlation between average CD and CP metrics across all target regions between HCP and BLS cohort. Additionally, after ranking all target regions by their average CD and CP values in descending order for each cohort, the resulting rankings were correlated using Spearman correlation.

Abbreviations: CD = connection density; contral. = contralateral; CP = connection probability.

### 3.3 Control Analysis for Confounding Effects of Claustrum Surrounding Grey Matter Connectivity

Claustrum tractography is notoriously delicate due to the structure's thin shape, directly adjacent white matter, and neighboring grey matter structures. To test whether the probabilistic tractography results that we found were confounded by surrounding brain matter connectivity, specifically the directly adjacent putamen and insula, we performed a control analysis. This involved replicating probabilistic tractography to all target regions as we had done it for the claustrum but using putamen and insula masks as seed regions. After deriving the same connectivity measures, CD and CP, we fitted a linear regression model to determine if these metrics were independent of connectivity from these potentially confounding regions.

The regression model for each target region, containing putamen and insula connectivity metrics, explained a low to moderate portion of the variance in the claustrum connectivity metrics in the HCP cohort. Notably, connectivity from the insula significantly influenced CD and CP to most target ROIs except the associative cortices (all for CD, all but associative occipital cortex for CP). On the other hand, connectivity from the putamen only significantly influenced CD to the parietal associative cortex, anterior and posterior cingulate, and CP to those same regions in addition to the prefrontal associative cortex. Overall, on average across all target ROIs, for CD, the model containing connectivity from both the insula and the putamen explained 30.3% of the variance (avg. *R* ^2^ = 0.303, avg. Adjusted *R* ^2^ = 0.287). The model for CP explained 37.7% (avg. *R* ^2^ = 0.377, avg. Adjusted *R* ^2^ = 0.361).

For the BLS cohort, which served as a secondary comparison cohort, the model exhibited a higher explanatory power. On average, across all target regions, insula and putamen connectivity together explained 46.1% of the variance for both CD (avg. *R* ^2^ = 0.461, adj. *R* ^2^ = 0.447) and CP (avg. *R* ^2^ = 0.461, adj. *R* ^2^ = 0.447). In this cohort, there was a more pronounced influence of putamen connectivity compared to the HCP cohort, especially for CP. For CD, Putamen connectivity did not significantly influence the associative cortices, the primary somatosensory cortex or connectivity to any subcortical ROIs, while Insula connectivity did not influence the associative cortices except the prefrontal cortex as well as the primary visual cortex.

The results from all target regions are summarized in Tables 5 and 6 for the HCP cohort, and in Tables 7 and 8 for the BLS cohort.

**TABLE 5:** Control analysis on the influence of insula and putamen connectivity on claustrum CD across target ROIs, HCP cohort.

Target ROI	*t*‐test		Multiple regression analysis									
	*t*	*p*	Putamen				Insula				*R* ^2^	Adj. *R* ^2^
			*b*	Beta	*t*	*p*	*b*	Beta	*t*	*p*		
AC_Occ	36.63	< 0.001	0.106	0.213	1.96	0.053	0.100	0.202	1.86	0.066	0.096	0.073
AC_Par	26.08	< 0.001	0.148	0.405	3.90	< 0.001	0.020	0.113	1.09	0.279	0.197	0.177
AC_PFC	46.72	< 0.001	0.048	0.206	1.87	0.065	0.048	0.139	1.26	0.211	0.059	0.035
AC_Temp	35.09	< 0.001	0.017	0.018	0.155	0.877	0.039	0.217	1.92	0.059	0.049	0.024
C_ant	11.32	< 0.001	0.134	0.516	7.13	< 0.001	0.511	0.445	6.15	< 0.001	0.706	0.699
C_pos	17.54	< 0.001	0.110	0.441	4.69	< 0.001	0.227	0.409	4.35	< 0.001	0.593	0.583
PC_aud	13.38	< 0.001	0.882	0.151	1.53	0.131	0.064	0.454	4.58	< 0.001	0.236	0.217
PC_mot	18.37	< 0.001	−0.770	−0.200	−2.14	0.036	0.265	0.605	6.48	< 0.001	0.353	0.336
PC_sens	21.84	< 0.001	0.037	0.185	1.65	0.104	0.022	0.072	0.639	0.525	0.036	0.011
PC_vis	26.35	< 0.001	0.161	0.180	1.74	0.086	0.495	0.392	3.78	< 0.001	0.225	0.205
SC_BG_Pal	21.86	< 0.001	—	—	—	—	0.165	0.454	4.53	< 0.001	0.207	0.196
SC_BG_SN	12.50	< 0.001	0.039	0.128	1.23	0.223	0.783	0.428	4.11	< 0.001	0.234	0.215
SC_Hip	24.63	< 0.001	0.006	0.074	0.751	0.455	0.286	0.567	5.75	< 0.001	0.345	0.345
SC_NM_Dop	15.43	< 0.001	0.052	0.264	2.86	0.005	0.671	0.548	5.93	< 0.001	0.524	0.511
SC_NM_LC	14.96	< 0.001	0.058	0.188	1.64	0.105	0.959	0.587	5.13	< 0.001	0.544	0.532
SC_NM_R	17.11	< 0.001	0.037	0.197	1.86	0.067	0.656	0.492	4.63	< 0.001	0.389	0.373
SC_Thal	27.00	< 0.001	0.019	0.204	1.97	0.053	0.114	0.473	4.56	< 0.001	0.360	0.344
Means											**0.303**	**0.287**
SD											**0.200**	**0.206**

*Note:* The model containing connectivity from both the insula and the putamen explained 30.3% of the variance (avg. *R* ^2^ = 0.303, avg. Adjusted *R* ^2^ = 0.287). Data is shown for left side ipsilateral connectivity on HCP cohort. See Table S10 in Supporting Information for information on right side ipsilateral connectivity.

Abbreviations: HCP = human connectome project; ROI = region of interest; SD = standard deviation.

**TABLE 6:** Control analysis on the influence of insula and putamen connectivity on claustrum CP across target ROIs, HCP cohort.

Target ROI	*t*‐test		Multiple regression analysis									
	*t*	*p*	Putamen				Insula				*R* ^2^	Adj. *R* ^2^
			*b*	Beta	*t*	*p*	*b*	Beta	*t*	*p*		
AC_Occ	43.83	< 0.001	0.230	0.068	0.700	0.486	2.45	0.504	5.17	< 0.001	0.265	0.246
AC_Par	37.64	< 0.001	0.839	0.432	4.18	< 0.001	0.058	0.036	0.347	0.729	0.193	0.172
AC_PFC	108.4	< 0.001	0.346	0.435	4.31	< 0.001	0.447	0.193	1.91	0.060	0.211	0.191
AC_Temp	45.17	< 0.001	0.496	0.086	0.769	0.444	0.355	0.199	1.78	0.079	0.053	0.029
C_ant	9.884	< 0.001	0.783	0.459	6.84	< 0.001	4.81	0.529	7.89	< 0.001	0.742	0.742
C_pos	17.25	< 0.001	0.585	0.415	4.40	< 0.001	1.69	0.439	4.65	< 0.001	0.606	0.596
PC_aud	14.43	< 0.001	2.42	0.177	1.79	0.078	0.153	0.445	4.49	< 0.001	0.238	0.218
PC_mot	17.77	< 0.001	0.002	0.002	0.024	0.981	1.63	0.677	7.97	< 0.001	0.458	0.444
PC_sens	24.01	< 0.001	0.251	0.221	2.08	0.041	0.583	0.280	2.63	0.010	0.118	0.096
PC_vis	26.81	< 0.001	0.481	0.083	0.870	0.388	5.22	0.547	5.71	< 0.001	0.329	0.312
SC_BG_Pal	22.25	< 0.001	—	—	—	—	0.546	0.456	4.18	< 0.001	0.318	0.300
SC_BG_SN	12.13	< 0.001	0.046	0.137	1.33	0.187	0.901	0.445	4.33	< 0.001	0.254	0.235
SC_Hip	24.69	< 0.001	0.034	0.089	1.04	0.302	1.77	0.682	8.00	< 0.001	0.520	0.508
SC_NM_Dop	14.62	< 0.001	0.082	0.293	3.17	0.002	0.985	0.529	5.73	< 0.001	0.538	0.526
SC_NM_LC	13.36	< 0.001	0.063	0.186	1.66	0.100	1.06	0.604	5.29	< 0.001	0.568	0.557
SC_NM_R	15.85	< 0.001	0.068	0.226	2.21	0.030	1.16	0.519	6.09	< 0.001	0.454	0.440
SC_Thal	28.35	< 0.001	0.107	0.212	2.42	0.018	0.960	0.609	6.91	< 0.001	0.544	0.532
Means											**0.377**	**0.361**
SD											**0.193**	**0.199**

*Note:* The model containing connectivity from both the insula and the putamen explained 37.7% of the variance (avg. *R* ^2^ = 0.377, avg. Adjusted *R* ^2^ = 0.361). Data is shown for left side ipsilateral connectivity on HCP cohort. See Table S11 in Supporting Information for information on right side ipsilateral connectivity.

Abbreviations: HCP = human connectome project; ROI = region of interest; SD = standard deviation.

**TABLE 7:** Control analysis on the influence of insula and putamen connectivity on claustrum CD across target ROIs, BLS cohort.

Target ROI	*t*‐test		Multiple regression analysis									
	*t*	*p*	Putamen				Insula				*R* ^2^	Adj. *R* ^2^
			*b*	Beta	*t*	*p*	*b*	Beta	*t*	*p*		
AC_Occ	16.32	< 0.001	0.097	0.328	2.83	0.006	0.226	0.347	2.98	0.004	0.374	0.357
AC_Par	22.09	< 0.001	0.079	0.333	3.17	0.002	0.049	0.273	2.60	0.011	0.250	0.231
AC_PFC	46.81	< 0.001	0.014	0.082	0.783	0.436	0.099	0.368	3.49	< 0.001	0.150	0.128
AC_Temp	25.93	< 0.001	0.146	0.272	2.62	0.011	0.079	0.025	3.20	0.002	0.240	0.221
C_ant	10.83	< 0.001	0.139	0.415	4.52	< 0.001	0.449	0.496	5.41	< 0.001	0.735	0.728
C_pos	10.79	< 0.001	0.138	0.415	4.11	< 0.001	0.321	0.378	3.74	< 0.001	0.508	0.496
PC_aud	10.71	< 0.001	1.12	0.425	4.92	< 0.001	0.068	0.429	4.96	< 0.001	0.447	0.433
PC_mot	11.49	< 0.001	0.048	0.368	4.06	< 0.001	0.283	0.438	4.84	< 0.001	0.447	0.433
PC_sens	14.94	< 0.001	0.027	0.225	2.62	0.011	0.217	0.598	6.94	< 0.001	0.518	0.506
PC_vis	9.24	< 0.001	0.541	0.473	4.12	< 0.001	0.144	0.265	2.31	0.023	0.468	0.454
SC_BG_Pal	21.84	< 0.001	—	—	—	—	0.566	0.572	6.20	< 0.001	0.327	0.319
SC_BG_SN	10.86	< 0.001	0.069	0.192	2.17	0.033	1.58	0.586	6.62	< 0.001	0.453	0.439
SC_Hip	11.80	< 0.001	0.031	0.020	1.50	0.138	0.529	0.580	5.26	< 0.001	0.495	0.482
SC_NM_Dop	12.25	< 0.001	0.035	0.194	2.28	0.025	1.28	0.621	7.31	< 0.001	0.508	0.496
SC_NM_LC	10.74	< 0.001	0.057	0.222	3.33	0.001	2.14	0.728	10.9	< 0.001	0.739	0.733
SC_NM_R	10.60	< 0.001	0.046	0.244	2.96	0.004	1.11	0.618	7.50	< 0.001	0.577	0.566
SC_Thal	16.54	< 0.001	0.033	0.220	2.71	0.008	3.44	0.644	7.91	< 0.001	0.593	0.582
Means											**0.461**	**0.447**
SD											**0.160**	**0.163**

*Note:* The model containing connectivity from both the insula and the putamen explained 46.1% of the variance (avg. *R* ^2^ = 0.461, avg. Adjusted *R* ^2^ = 0.447). Data is shown for left side ipsilateral connectivity on BLS cohort. See Table S12 in Supporting Information for information on right side ipsilateral connectivity.

Abbreviations: BLS = Bavarian longitudinal study; ROI = region of interest; SD = standard deviation.

**TABLE 8:** Control analysis on the influence of insula and putamen connectivity on claustrum CP across target ROIs, BLS cohort.

Target ROI	*t*‐test		Multiple regression analysis									
	*t*	*p*	Putamen				Insula				*R* ^2^	adj. *R* ^2^
			*b*	Beta	*t*	*p*	*b*	Beta	*t*	*p*		
AC_Occ	17.43	< 0.001	0.491	0.228	1.93	0.057	4.01	0.440	3.72	< 0.001	0.376	0.360
AC_Par	28.08	< 0.001	0.516	0.351	3.70	< 0.001	0.614	0.394	4.15	< 0.001	0.359	0.342
AC_PFC	86.35	< 0.001	0.212	0.266	2.45	0.017	0.301	0.146	1.34	0.183	0.105	0.082
AC_Temp	31.46	< 0.001	0.896	0.381	2.35	0.021	0.636	0.284	2.68	0.009	0.179	0.158
C_ant	12.14	< 0.001	0.731	0.447	4.64	< 0.001	2.29	0.449	4.56	< 0.001	0.690	0.682
C_pos	10.36	< 0.001	0.710	0.394	4.31	< 0.001	2.64	0.472	5.17	< 0.001	0.617	0.607
PC_aud	11.49	< 0.001	2.52	0.404	4.61	< 0.001	0.168	0.434	4.94	< 0.001	0.430	0.415
PC_mot	11.49	< 0.001	0.188	0.332	4.00	< 0.001	1.81	0.542	6.54	< 0.001	0.543	0.531
PC_sens	14.64	< 0.001	0.144	0.192	2.49	0.015	1.94	0.687	8.92	< 0.001	0.622	0.612
PC_vis	8.48	< 0.001	3.64	0.474	4.08	< 0.001	11.0	0.246	2.12	0.037	0.446	0.432
SC_BG_Pal	21.05	< 0.001	—	—	—	—	1.72	0.534	5.61	< 0.001	0.285	0.276
SC_BG_SN	10.02	< 0.001	0.085	0.208	2.30	0.024	1.71	0.553	6.11	< 0.001	0.421	0.406
SC_Hip	11.86	< 0.001	0.190	0.233	2.11	0.038	2.35	0.529	4.78	< 0.001	0.505	0.493
SC_NM_Dop	11.43	< 0.001	0.047	0.192	2.21	0.030	1.68	0.611	7.05	< 0.001	0.498	0.485
SC_NM_LC	8.26	< 0.001	0.095	0.338	4.26	< 0.001	2.07	0.565	7.11	< 0.001	0.591	0.580
SC_NM_R	8.42	< 0.001	0.102	0.338	4.17	< 0.001	1.58	0.551	6.79	< 0.001	0.568	0.556
SC_Thal	18.82	< 0.001	0.221	0.309	3.81	< 0.001	1.75	0.579	7.14	< 0.001	0.594	0.983
Means											**0.461**	**0.447**
SD											**0.161**	**0.165**

*Note:* The model containing connectivity from both the insula and the putamen explained 46.1% of the variance (avg. *R* ^2^ = 0.461, avg. Adjusted *R* ^2^ = 0.447). Data is shown for left side ipsilateral connectivity on BLS cohort. See Table S13 in Supporting Information for information on right side ipsilateral connectivity.

Abbreviations: BLS = Bavarian longitudinal study; ROI = region of interest; SD = standard deviation.

#### 3.3.1 Tractography Across Hemispheres

To further examine the reliability of probabilistic tractography in detecting streamline connectivity of the claustrum, we also performed probabilistic tractography from the claustrum to all target ROIs of the contralateral hemisphere. Three target ROIs, namely the raphe nuclei, the dopaminergic nuclei, and the cholinergic basal forebrain, are non‐pair regions, meaning strictly contralateral reconstruction is not meaningful. Therefore, we excluded these three regions in this analysis. Proximity was not an issue limiting robust streamline reconstruction here, so tracking to the contralateral anterior and posterior insula, amygdala, and striatum was possible.

**FIGURE 7:**
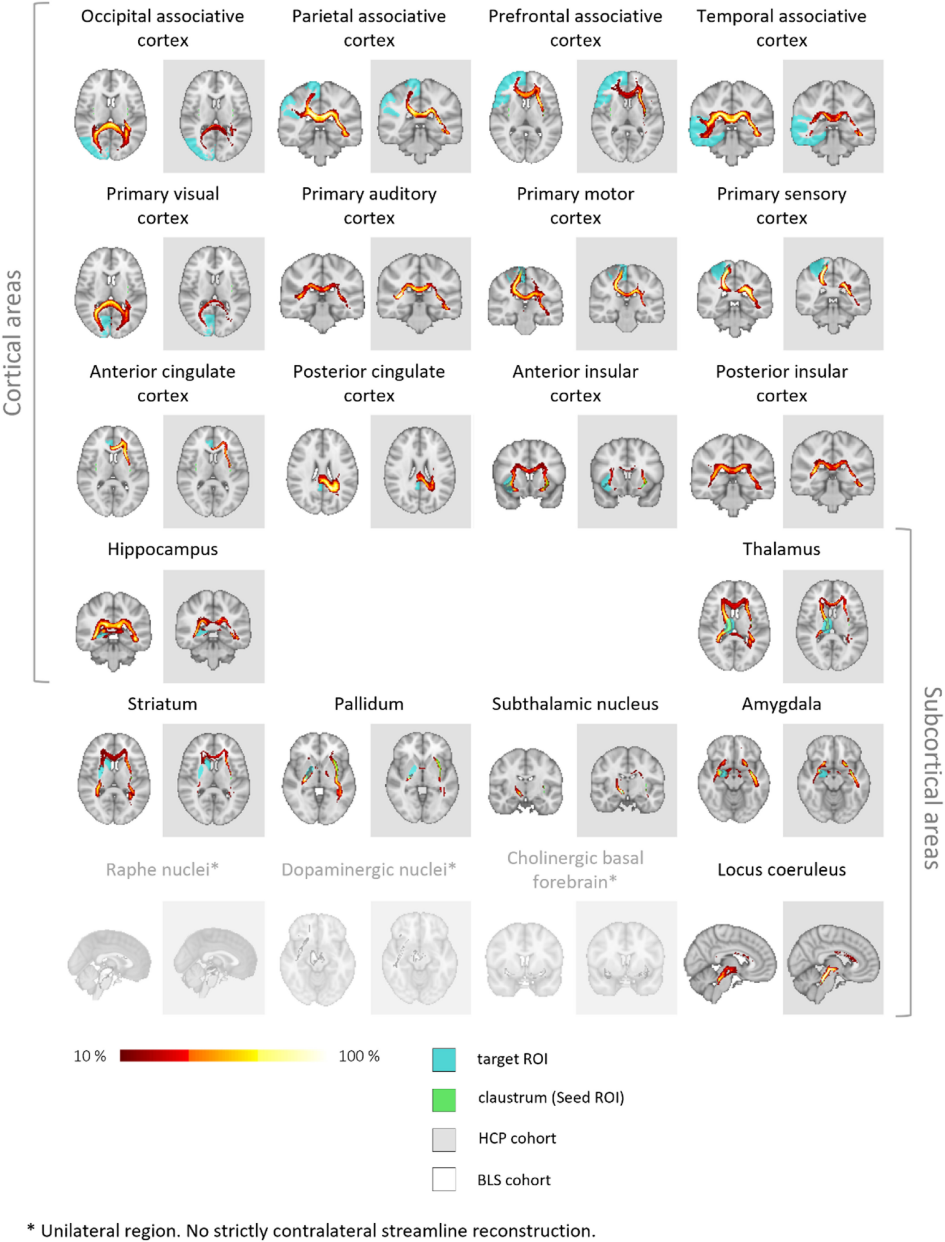
Contralateral claustrum connectivity: Contralateral streamline, comparison across cohorts. The cohort‐averaged reconstructed streamline tracts, depicting in each voxel the percentage of subjects having their individual streamline tract passing through that voxel, are displayed overlaid on the MNI‐152 T1 2mm brain. Only the reconstructions from the left‐side claustrum to the right‐side (contralateral) ROIs are shown. The representation is thresholded at 10% (lower threshold). BLS = Bavarian longitudinal study; HCP = human connectome project; ROI = region of interest.

**FIGURE 8:**
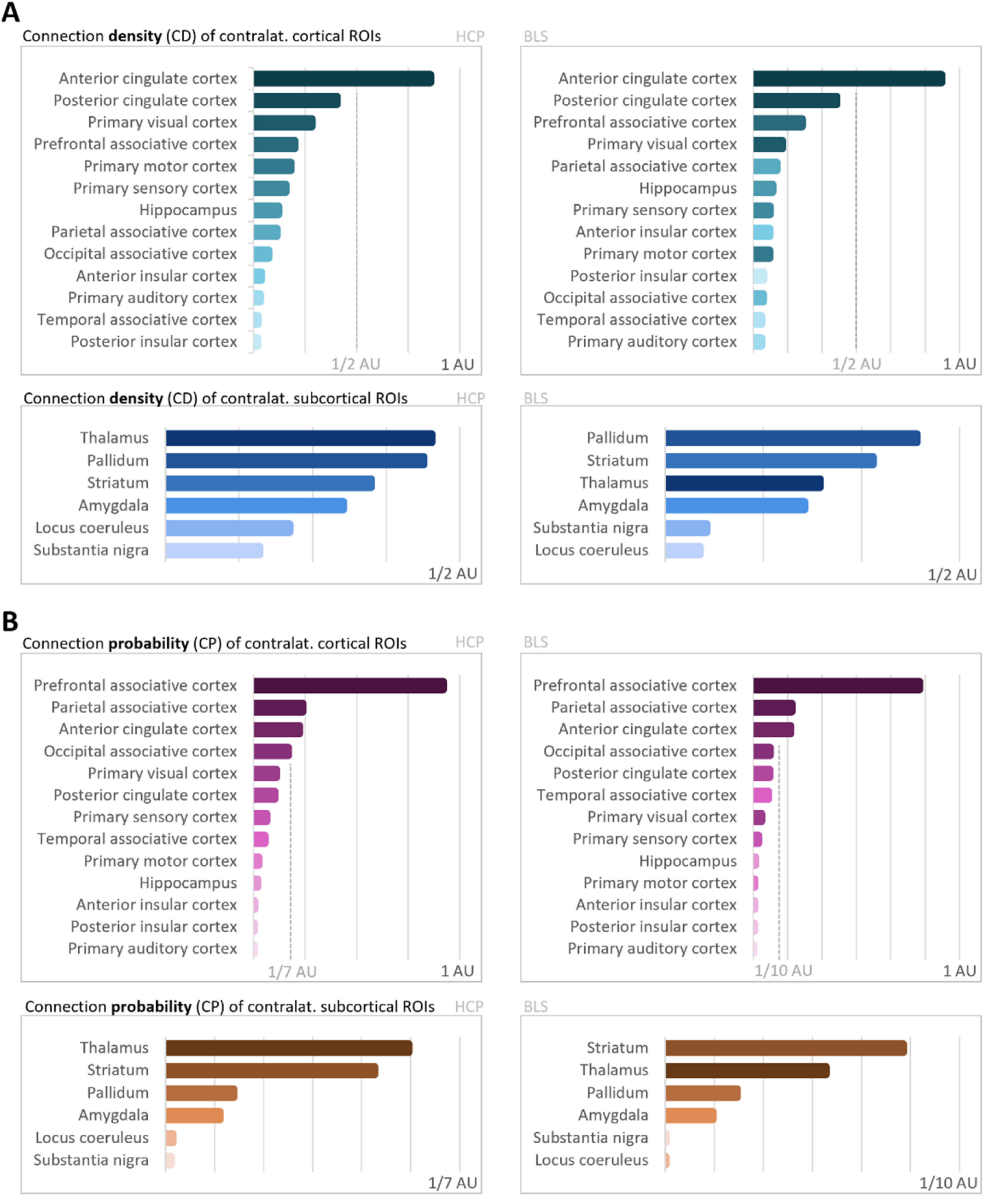
Contralateral claustrum connectivity: Outcomes across cohorts. (A) Connection density of contralateral connectivity to cortical and subcortical ROIs for HCP (left) and BLS (right) cohorts. (B) Connection probability of contralateral connectivity to cortical and subcortical ROIs for HCP (left) and BLS (right) cohorts. Metrics are shown for cortical and subcortical areas separately, and for the HCP cohort (left side) as well as the BLS cohort (right side). As connectivity metrics are at most meaningful in order to compare reconstructions done in the same way on identical image characteristics, only arbitrary units are displayed. AU = arbitrary unit; BLS = Bavarian longitudinal study; CD = connection density; contral. = contralateral; CP = connection probability; HCP = human connectome project; ROI = region of interest.

The reconstructed streamline tracts, depicting in each voxel the percentage of subjects having their individual streamline tract passing through that voxel, are shown in Figure 7. The representation is thresholded at 10%. Similarly, to the ipsilateral regions, after across‐cohort averaging, we found anatomically plausible streamline reconstructions with tract‐like morphology, although significantly more dispersed, or not as consistent across subjects. To compare the connectivity patterns to subcortical areas revealed by relative CD and CP values, we also visualized them side‐by‐side in Figure 8. For cortical ROIs, CD and CP show some more marked differences, although the overall divisions between regions with high and regions with low relative values are preserved. For subcortical ROIs, the order of connectivity is largely identical, except the pallidum ranks lower by its CP than by its CD. We conclude that, by visual examination of all individual subjects and of the across‐cohort averages, streamline reconstruction is also possible to most contralateral regions, although slightly less robust than to ipsilateral regions.

### 3.4 Characterizing Claustrum Connectivity as Shown by Probabilistic Tractography

#### 3.4.1 Ipsilateral Claustrum Connectivity: Overall Greater Connection Density and Probability to Cortical Regions Compared to Subcortical Targets

Based on the above demonstrated robustness of DWI‐based tractography of claustrum connections across individuals, regions, scanners, and sequences, as well as hemispheres, we wanted to highlight some properties of DWI‐based claustrum connections. In order to quantitatively examine the structural connectivity of the claustrum, we computed two measures, CP and CD. We found that, in the HCP reference dataset, the highest density of connections to cortical areas, as reflected by the CD metric, was to the primary auditory cortex, followed by the primary visual cortex and then all associative cortices. Of those, CD was overall similar, with the occipital associative cortex being the least densely connected to the claustrum of all four. Somewhat surprisingly, we found the lowest CD to the posterior and anterior cingulate cortices, which had been identified as strongly connected to the claustrum in animal models (White and Mathur 2018; Chia, Augustine, and Silberberg 2020). For subcortical areas, we identified the pallidum and thalamus as being most densely connected, with locus coeruleus and the subthalamic nucleus being least densely connected (Figure 6, first half; Table S3).

Connection probability, overall, followed a similar pattern. For cortical areas, we found the highest probability of streamlines seeded from the claustrum reaching a particular target ROI for the associative cortices, with the prefrontal associative cortex having the highest CP; followed by temporal, parietal, and then occipital associative cortices. The lowest CP was found for the posterior cingulate cortex, hippocampus, primary motor cortex, and anterior cingulate cortex. For subcortical areas, CP was highest to the thalamus by far, followed by the pallidum. CD and CP follow the same pattern for subcortical areas (Figure 6, lower half; Table S3). Overall, we conclude that streamline connectivity was denser and more probable to cortical areas than subcortical, the thalamus, and the pallidum being notable exceptions and surpassing some subcortical areas.

#### 3.4.2 Contralateral Claustrum Connectivity: Lower Density Than Ipsilateral Targets, Balanced Representation Across Cortical and Subcortical Regions

Using the same procedure as for ipsilateral connectivity, we quantified contralateral connectivity using both CP and CD to examine the relative strength of the reconstructed connections. An overview of relative CDs and CPs can be found in Figure 8 (see Table S4 in Supporting Information for detailed streamline data). We found that the highest CD to cortical areas was, by far, to the cingulate cortices, with the streamline connections to the anterior cingulate cortex being significantly denser than to the posterior cingulate cortex. We conclude that, in contrast to ipsilateral connectivity, the contralateral associative cortices were overall a lot less connected to the claustrum, with limbic and primary cortices taking the lead. For subcortical ROIs, mirroring ipsilateral connectivity patterns, CD was strongest to the thalamus and the pallidum, and weakest to the substantia nigra.

Looking at CP, we found a slightly different pattern; the prefrontal associative cortex had by far the highest probability, followed by the parietal associative cortex and anterior cingulate cortex. The insular cortices and the primary auditory cortex had the lowest CP. For subcortical ROIs, CP was highest to the thalamus and the striatum, and again weakest to the substantia nigra.

Overall, we observed that contralateral subcortical targets were not significantly less densely or probably connected with the claustrum, as was the case when looking at ipsilateral connectivity; rather, the thalamus and pallidum were second only to the anterior cingulate cortex regarding CD. We conclude that streamline connectivity as revealed by probabilistic tractography provides robust evidence for widespread contralateral connectivity of the claustrum, which is nonetheless less pronounced than to ipsilateral targets.

## 4 Discussion

Although the extraordinarily high level of claustrum connectivity in the human brain is known, it has been unclear whether DWI‐based tractography is a robust tool to study aspects of claustrum connectivity in humans. The current study examines consistency and replicability of DWI‐based tractography of claustrum connections with both cortical and subcortical regions for different streamline connection properties in 162 young healthy controls equally distributed across two large cohorts. Using an automated claustrum segmentation protocol combined with probabilistic tractography in two cohorts, we investigated the ipsilateral and contralateral connectivity of the claustrum in terms of streamline‐based connection density and probability. We found DWI‐based tractography to be reliable in detecting human claustrum connections across individuals and regions, both cortical and subcortical, across differing DWI protocols and scanners, as well as across hemispheres. Reconstruction using this method was possible to most cortical targets except nearby regions such as ipsilateral insular cortices or the striatum. Regarding connections of the claustrum, we found widespread ipsilateral connectivity favoring cortical targets, and less dense, but extensive, contralateral connectivity of the claustrum. Thus, this study demonstrates—to the best of our knowledge for the first time—robust DWI‐based in vivo detection of claustrum connections in humans of different cohorts with various scanners and scanning parameters. This result suggests that DWI‐tractography might be a reliable tool for studying claustrum connectivity in the human brain.

### 4.1 Human Claustrum Connections as Detected by DWI‐Based Tractography Are Consistent and Replicable Across Individuals, Regions, Acquisition Methods, and Hemispheres

#### 4.1.1 Consistency Across Individuals and Regions

We found robust streamline reconstruction by probabilistic tractography to be possible and anatomically plausible to all primary, associative, and limbic cortices as well as subcortical regions (Figures 2, 3, and 6, Table 2). No meaningful results could be obtained for regions in too close proximity to the claustrum. This included, ipsilaterally, the anterior and posterior insula, the cholinergic basal forebrain, the amygdala, and the striatum, even with the high resolution of diffusion‐weighted imaging provided by the HCP cohort. Further studies using diffusion‐weighted imaging‐based tractography to examine the connectivity of the claustrum should take this limitation into account. This is especially important when employing a high‐yield, whole‐brain approach minimizing human intervention at the analysis stage, in order to prevent false‐positive results implicating those regions in high proximity.

#### 4.1.2 Replicability Across Sequences and Scanners/Cohorts, Independence From Surrounding Grey Matter Connectivity and Connectivity Metric Stability

##### 4.1.2.1 Replicability

To determine the reproducibility of our findings, we assessed the robustness of claustrum streamline reconstructions across different samples and imaging sequences. To this end, we replicated all streamline reconstructions executed in our widely accepted and used reference cohort, the HCP cohort, in an independent cohort, the BLS cohort. We found that cohort average streamline tracts were highly similar anatomically, with a slightly larger dispersion across the BLS cohort but a moderately high volumetric overlap as quantified by an average DC of 0.59 and 0.61 for L and R sides, respectively (for ipsilateral streamline maps) across all examined regions (Table 3).

In addition, we quantified between‐cohort robustness by performing voxel‐wise volumetric correlations for both of the cohort‐average maps and across all individual subjects both within‐ and across‐cohort (Figure 5). We found a high correlation between cohort‐average tract maps of 0.77 and 0.78 for the left and right sides. Across‐cohort comparison across subject pairs gave an average correlation of 0.291 and 0.311 for the left and right sides, respectively, significantly lower than within‐cohort comparison (0.366 and 0.360 for left side within‐cohort for HCP and BLS, respectively). These results are in line with findings from Warrington and colleagues, who also reported a higher correlation within cohorts (0.51 for HCP and 0.54 for UK Biobank) compared to between cohorts (0.41) (Warrington et al. 2020). Although our correlation values are slightly lower by 0.1–0.15 on average, this consistency suggests that our observed patterns are standard. The similar pattern of higher within‐cohort correlations indicates that despite some individual variability, there is a general consistency in the spatial distribution of tracts within the same cohort. At the same time, between‐cohort differences likely reflect variations in data quality, scanner, and demographic factors.

Finally, we also compared the quantification of streamline reconstructions across our two cohorts for CD and CP (see Figure 6 and Table 4). The scanning differences between the HCP and BLS cohorts were considerable, most notably with the acquisition of multi‐shell data and 90 diffusion‐weighted directions for the HCP cohort compared to BLS's single‐shell information with 32 directions. Nonetheless, the pattern of relative CD and CP was nearly identical for subcortical regions (Figure 6A,B, lower panels respectively), very similar for CP of cortical regions (Figure 6B, upper panel), and slightly diverging for CD (Figure 6A, upper panel). This was reflected in the correlation values of these metrics: we saw a more moderate and non‐significant correlation between CD of the two cohorts (*r* = 0.635, *p* = 0.006 and *r* = 0.466, *p* = 0.059 for L and R sides), but a strong and highly significant correlation for CP (*r* = 0.980, *p* < 0.001 and *r* = 0.981, *p* < 0.001 for L and R sides). The main difference here was that the primary auditory and visual cortices within the BLS cohort were not more densely connected to the claustrum than the associative cortices. One possible explanation is that the BLS cohort's simpler acquisition scheme rendered the higher‐order modelling of crossing fibers during tractography not possible, and so probabilistic tractography was not as accurate in detecting or resolving these particular connections. Nonetheless, when correlating the rankings of each target region by CD and CP, we found a strong and highly significant correlation for both metrics, suggesting that overall, relative connectivity of the claustrum was well replicated in the second cohort.

##### 4.1.2.2 Surrounding Grey Matter Confounds

While testing the independence of the claustrum connectivity metrics (CD and CP) from the connectivity of the directly neighboring gray matter regions, the putamen and the insula, we found significant influence mainly of insula connectivity on CD and CP to most target ROIs. Meanwhile, putamen connectivity statistically significantly influenced only a few regions. Overall, 30.3% of the variance in CD and 37.7% of the variance in CP from the claustrum across all target ROIs could be explained by the connectivity of the potentially confounding regions.

Our results indicate that the influence of neighboring regions varies significantly by target region. Specifically, connections between the claustrum and regions such as the anterior and posterior cingulate, hippocampus, dopaminergic nuclei, locus coeruleus, and thalamus are more susceptible to contamination from the insula and putamen. Connectivity metrics to the anterior and posterior cingulate, in particular, stand out as strongly influenced by both the insula and the putamen. In contrast, connections to the associative cortices showed relative independence from these neighboring regions, suggesting that tractography results for these areas are less affected by confounding influences. This highlights the importance of considering the potential impact of surrounding brain matter when interpreting tractography results, especially for certain target regions. Considering these results, probabilistic tractography seems a robust choice for examining claustrum connectivity to the associative cortices. However, further adaptations are likely needed to confirm connectivity to most subcortical regions and the cingulate cortices.

The control analysis in the second cohort, the BLS cohort, revealed an overall similar pattern, particularly regarding high contamination of connectivity to the anterior and posterior cingulate cortices and a lower level of influence of the putamen across all regions. Interestingly, a larger influence of putamen connectivity was found in this cohort, particularly for CP. On average, across all regions, 16% more variance in CD (30.3% in HCP, 46.1% in BLS) and 8% more variance in CP (37.7% in HCP, 46.1% in BLS) could be explained by neighboring region connectivity in the BLS cohort. The likely explanation for this increase lies in the differences in imaging protocols; the higher resolution and greater number of diffusion‐weighted directions in the HCP cohort possibly allowed for better separation of connections between neighboring regions.

Nonetheless, the influence of neighboring regions extends beyond claustrum connectivity alone and is a general concern in probabilistic tractography (Behrens et al. 2007). This method yields probabilistic maps of the most likely path of white matter connections without inherent attempts at showing one singular truth per seed region or seed voxel (Behrens et al. 2007; Soares et al. 2013). Matters are further complicated by the fact that white matter at the resolution of the MRI images used for probabilistic tractography can, of course, contain fibers originating from different brain regions (Jbabdi and Johansen‐Berg 2011; Sotiropoulos and Zalesky 2019), further complicating the interpretation of overlapping results between neighboring source regions which may be connected to the same target regions. Thus, although we provide one method to investigate potential contamination of our reported connectivity values, developing robust methods to control and potentially remedy the influence of these confounding regions should be the subject of a separate study. This would involve testing various methods and studying their implications on the outputs of probabilistic tractography and their validity, which goes beyond the scope of the current study.

##### 4.1.2.3 Metric Stability

While many recent studies favor a metric like CD (Gu et al. 2021) because it is normalized by the total volume of connected regions and allows for more straightforward comparability across regions of varying sizes, CP was the metric of choice for a long time. It is still sometimes employed (Cao et al. 2013), and was previously reported to be stable between different approaches of modeling crossing fibers (Behrens, Johansen‐Berg, et al. 2003; Behrens et al. 2007). In line with this, the CP metric was very robust between our cohorts. A possible consequence could be to opt for the CD metric when examining or comparing individuals stemming from the same cohort, such as analyzing differences between the preterm and the term‐born groups within the BLS cohort, while utilizing the CP metric when investigating individuals from different cohorts and different scanning parameters.

#### 4.1.3 Reliability Across Hemispheres

Examining the reliability of probabilistic tractography in tracing streamline connections to the opposite hemisphere, we found streamline reconstruction to contralateral areas to be possible and robust across subjects. However, there was a higher divergence of the topography of streamline tracts between individuals than we observed for ipsilateral regions, stemming from a lower agreement across subjects on the anatomy of the streamline tract. This can be explained by the fact that inter‐hemispheric distances are, on average, longer than intra‐hemispheric ones (Roberts et al. 2016). Distance bias describes the fact that the number of streamlines that are reconstructed are influenced negatively by longer distances separating the seed and target region. This effect is inherent to probabilistic tractography due to the dispersion of the propagating streamlines in each step adding up along longer distances (Liptrot, Sidaros, and Dyrby 2014). Nonetheless, our cohort average maps show that reconstructions were still consistent across individuals for all examined regions, indicating that DWI‐based tractography can reliably detect even longer streamlines. This caveat should be kept in mind when directly comparing streamline metrics for ipsi‐ and contralateral areas, as they may be impacted by the simple fact that they are further away from the seed region.

### 4.2 Our Automated Approach to Claustrum Segmentation Reduces Bias and Enhances Precision

To precisely reconstruct streamlines stemming from the claustrum for each subject, we used a deep‐learning‐based automated claustrum segmentation tool (Li et al. 2021) to segment bilateral claustra (Figure 1). We found this method to be highly precise in delimiting the claustrum in both cohorts, as well as largely more time‐efficient when compared to manual segmentation, needing only sporadic adjustments. This is particularly important in the claustrum, a region so small, irregular, and surrounded by regions that could easily act as potential confounders for tractography. It requires both, a high accuracy and a good level of experience, to identify and delimit it accurately. Manual annotations, even by experienced neuroradiologists, can differ significantly between annotators, making consistency paramount to obtain reliable streamline reconstructions (Billot et al. 2020; Li et al. 2021; Neubauer et al. 2022). Other studies have employed the widely used technique of using a volume averaged across subjects as a seed region. As just mentioned, in the case of the claustrum, a deviation of 1–2 voxels could mean seed voxels placed within the external and extreme capsule or even the striatum or the insula. This inexactitude is problematic and could result in a high number of false positive results not pertaining to the claustrum itself as the source region.

Maybe most importantly, this method can be applied to a large number of subjects, requiring only the presence of a T1w structural MRI scan (typically acquired in most imaging studies or routine scans), and will allow for easy reproducibility of our findings and transfer to the study of different pathologies. To date, it has already been applied successfully to segment the claustra of preterm‐born neonates after transfer learning (Neubauer et al. 2023).

### 4.3 Probabilistic Tractography Reveals Widespread Ipsilateral Connectivity Favoring Cortical Targets, and Less Dense, but Extensive, Contralateral Connectivity of the Claustrum

#### 4.3.1 Ipsilateral Connectivity of the Claustrum

Our investigation into the ipsilateral connectivity of the claustrum revealed a multifaceted, widespread network of streamline connections and provided new, interesting insights into relative connectivity to the different areas.

Overall, CD to cortical areas was higher than to subcortical regions; surprisingly, we observed the highest CDs to the primary auditory and visual cortices, followed by the associative cortices. A cortical connectivity favoring the associative cortices, especially the prefrontal and temporal associative cortices, has been widely described previously, both in humans (Torgerson and van Horn 2014; Milardi et al. 2015; Torgerson et al. 2015) and in animals (Goll, Atlan, and Citri 2015; Wang et al. 2017; Nikolenko et al. 2021). Notably, the pattern of connectional hierarchy that we observed for the associative cortices—with the prefrontal and temporal cortices exhibiting highest connectivity to the claustrum, followed by parietal, and then occipital—is in agreement with the pattern suggested by Torgerson et al. (2015). Adding to this, we found strong streamline connections to the thalamus and the putamen for subcortical areas, as has previously been described in animal models (Dillingham et al. 2017; Nikolenko et al. 2021).

Meanwhile, our finding of the highest CD to primary auditory and visual cortices was unexpected; to date, the claustrum has mainly been described as being, at best, minimally connected with sensory areas (Wang et al. 2017; Jackson, Smith, and Lee 2020; Nikolenko et al. 2021). Nonetheless, Milardi et al. (2015) reported connections to auditory and visual cortices, although their relative strength was not quantified. Furthermore, strong visual input to the claustrum is known in cats (LeVay and Sherk 1981), while Remedios and colleagues as well as Coates and colleagues describe responses to audio‐visual stimuli within the claustrum (Remedios, Logothetis, and Kayser 2010; Coates et al. 2023). Our results would be consistent with a potential role of the claustrum in integrating visual and auditory sensory information in humans, and further studies should be done to elucidate the nature of these connections and their functional implications in humans.

Another initially surprising finding was the very low CD to the ipsilateral cingulate cortices, particularly the anterior cingulate cortex (ACC), which was suggested to be densely and reciprocally connected to the claustrum, specifically in the rat and mouse (Wang et al. 2017; White and Mathur 2018; Krimmel et al. 2019; Wang et al. 2023). However, recent studies provide a nuanced view. Koga et al. (2024) identified projections from the ACC to the contralateral claustrum in a mouse model through the use of viral tracers. Additionally, Wang et al. (2017) also found connections from the claustrum to be denser to the contralateral cingulate cortices than the ipsilateral onces. Remarkably, we found exactly the same pattern of strong claustrum connectivity to the *contralateral* cingulate cortices in our samples. Our results using diffusion imaging and probabilistic tractography thus reflect findings in mice carried out by viral tracer. Further studies utilizing DWI in non‐human primates should be useful and may serve to further elucidate differences and communalities across species. This also gives further evidence that human DWI‐based tracking studies can reflect tracer‐based studies in mice, and that DWI can be used in non‐human primates to compare differences across species.

#### 4.3.2 Contralateral Connectivity of the Claustrum

Our exploration of contralateral connectivity of the claustrum revealed distinct patterns in comparison to ipsilateral connectivity and provided further evidence for widespread contralateral connectivity of the claustrum in humans.

The quantitative results suggested that, overall, connections to the contralateral hemisphere were less dense than the ipsilateral hemisphere but consistently present and traceable. This is in agreement with previous findings in mice (Wang et al. 2017) and rats (White et al. 2017). Additionally, the claustrum had dense contralateral streamline connections to the anterior and posterior cingulate cortices (in stark contrast to ipsilateral connectivity), as well as to the thalamus and pallidum. This asymmetric organization pertaining to the cingulate cortices was also described in mice (Wang et al. 2017; Zingg et al. 2018). Meanwhile, the insular cortices, the primary visual and auditory cortices, and the associative cortices, except the prefrontal associative cortex, seemed sparsely connected.

These contralateral connections seemed to mainly pass through the corpus callosum, with some limited evidence of possible involvement of the anterior commissure. This has previously been suggested in studies both in humans (Arrigo et al. 2017) and animals (Park, Tyszka, and Allman 2012), albeit in much smaller samples. Overall, previous findings on patterns of contralateral connectivity of the claustrum are heterogeneous and sparse, making direct comparisons of our findings difficult.

An important factor in contralateral connectivity is that studies suggest claustro‐cortical projections to be denser to ipsilateral regions (with an existing, but weaker, contralateral projection), but with contralateral projections being stronger from the cortex to the claustrum (Mathur 2014; Jackson, Smith, and Lee 2020). This seems to be particularly true for frontal associative areas (Smith and Alloway 2010, 2014). Using probabilistic tractography, the directionality of connections cannot be deducted (Hahn et al. 2019). It may be that, in using this method, we cannot entirely do this complex pattern justice.

### 4.4 Strengths, Limitations, and Future Directions

Our study has several limitations that should be taken into account.

Regarding sample characteristics, we had a skewed gender distribution favoring male participants (56% in the HCP cohort, 64% in the BLS cohort), and perfect age and gender matching was not possible due to the limited number of participants in the BLS cohort. Additionally, the HCP cohort had a significantly larger age range of 13 years compared to BLS, which had a range of 3.3 years. As gender, but especially also age, impacts white matter (Kanaan et al. 2012; Lebel and Deoni 2018), these variations need to be considered when interpreting the results. Nonetheless, the highest rate of white matter changes happens in the first years of childhood and slows after reaching young adulthood (Lebel et al. 2012), which is the stage our participants were in.

The small size and irregular anatomy, as well as the location of the claustrum, make it a challenging target of study using imaging methods, particularly diffusion imaging, which inherently has a lower resolution and is prone to artifacts (Soares et al. 2013). Using a rigorous quality control protocol and an automated claustrum segmentation tool, we aimed to mitigate this issue and define each individual's claustrum as precisely as possible. Still, a heightened chance of false‐positive results remains.

An important consideration is the moderate agreement between datasets as quantified by the Dice coefficient. Although we observed high voxel‐wise correlations between cohorts, the DC values both for ipsilateral and contralateral connections suggest a moderate overlap in the range of 55%–60% on average across regions, depending on the hemisphere and laterality. This indicates variability in approximately 40% of the tract volume between the two datasets, likely reflecting the inherent differences in acquisition protocols, such as image resolution and the number of diffusion‐weighted directions. This variability again highlights the importance of considering acquisition parameters when designing future studies, particularly for “challenging” regions inherently prone to higher anatomical variability and/or smaller size. Notably, we also observed variances in DC values across different target regions, with for example primary cortices demonstrating more robust and consistent overlap, while other areas such as the hippocampus showed greater variability. This finding suggests that certain brain regions may be more sensitive to differences in imaging quality, reinforcing the need for tailored acquisition protocols depending on the exact connecting regions of interest. Nonetheless, it is important to consider that these DC values reflect comparisons of probabilistic maps, and that lower resolution and fewer diffusion directions tend to increase uncertainty and dispersion in tract reconstructions, yielding larger volumes of these probabilistic maps. More than just a volumetric disagreement between cohorts, these values could also reflect a higher precision of predictions in the HCP cohort when compared to the BLS cohort due to their more advanced protocols and resolution.

Probabilistic tractography, while being amongst the best techniques for studying white matter in vivo, cannot be regarded on the same level as directly visualizing axons. Indeed, the exact relationship between the primary output of probabilistic tractography, the count of streamlines connecting two regions, and the biological ground truth, is not entirely understood (Jones, Knösche, and Turner 2013). Notably, this number can be significantly influenced by several factors, an important one being the volume of the connected regions. To reduce this bias, we used connectivity metrics normalized by the total volume of connected regions (CD) or by the total number of streamlines seeded (CP) (Bajammal, Yoldemir, and Abugharbieh 2015). Nonetheless, probabilistic tractography, involving many steps, is inherently prone to accumulating errors and limited in allowing for direct biological interpretation (Jeurissen et al. 2019). Consequently, our findings can only provide approximations in the way of being the best approach we currently have to examine and describe anatomical connectivity non‐invasively in large human cohorts (Le Bihan and Johansen‐Berg 2012). The current trend in connectivity research is going towards larger and larger data sets with large‐scale network‐theoretical analysis methods (O'Donnell et al. 2019). Consequently, rigorous control of data and outputs to minimize false positives and incorporation of histologically obtained anatomical priors (Maier‐Hein et al. 2017), while time‐intensive, is needed. This is especially important in order not to lose sight of the object of study underneath the wealth of data and information that can be gleaned from a single MRI image. Further studies should aim to combine multiple methods of examining claustrum connectivity in humans, both in and ex vivo, to establish robust anatomical priors and investigate the correspondence of findings obtained through diffusion imaging with the biological ground truth.

Despite these limitations, our study exhibits significant strengths. The sample is large and spans two separate cohorts with distinct scanning parameters, encompassing a range from clinical routine to state‐of‐the‐art research use. This enhances the robustness and generalizability of the findings. Visual and quantitative quality checks were employed at each level of preprocessing, processing, and analysis to ensure high data quality and anatomical plausibility of the study's outcomes. Finally, we used an automated segmentation approach for individual, comparable, and high‐quality claustrum seed masks to enhance the accuracy of tractography. Collectively, these strengths support this study's findings, enhancing its applicability, reliability, and relevance in furthering our understanding of the claustrum's connectivity and the methods used for its exploration.

## 5 Conclusion

In conclusion, our study provides clear evidence for consistent and replicable DWI‐based tractography in detecting claustrum connections in humans across cortical and subcortical regions, diverse cohorts, scanners, and hemispheres. The streamline reconstructions demonstrated consistent anatomical plausibility, with reliable connectivity to various ipsilateral cortical and subcortical regions, favoring cortical targets. Contralateral connections, though less dense, were also consistently traceable, revealing distinct patterns compared to ipsilateral connectivity. The application of an automated claustrum segmentation tool further enhanced precision and reduced bias in streamline reconstructions. Despite challenges and limitations inherent to diffusion imaging, our findings suggest that DWI‐based tractography is a valuable tool for studying the complex connectivity of the claustrum in vivo, opening avenues for further exploration of its functional and pathophysiological implications in the human brain.

## Conflicts of Interest

The authors declare no conflicts of interest.

## Supporting information


**Data S1:** Supporting Information.

## Data Availability

The HCP young adult dataset used in this study is described on the humanconnectome webpage (https://www.humanconnectome.org/study/hcp‐young‐adult) and can be found on the online platform ConnectomeDB (https://db.humanconnectome.org; 100 Unrelated Sunjects subset of WU‐Minn HCP Data – 1200 Subjects release). BLS data used in this study are not publicly available but stored by the principal investigators of the Bavarian Longitudinal Study.
