# Supplementary material for: Human Claustrum Connections: Robust In Vivo Detection by DWI‐Based Tractography in Two Large Samples
Source: Hum Brain Mapp. 2024 Oct 13;45(14):e70042. doi: 10.1002/hbm.70042 (PMC11471578; doi:10.1002/hbm.70042)
Supplement: Supplementary file 1 — Data S1: Supporting Information. [file HBM-45-e70042-s001.docx]

**Supplementary material**

**Human claustrum connections: robust in-vivo detection by DWI-based tractography in two large samples**

**Table S1: Source atlas subregions used for generation of Target regions of interest (ROIs).** Numbers noted in square brackets for the subregions are the region’s numeric labels used within the labelling systems. Juelich and Harvard-Oxford cortical and subcortical atlases are available through FMRIB software library (FSL). All further atlases are available for download through their respective publications.

|  | Target | Atlas based on | Subregions, if multiple used |
| --- | --- | --- | --- |
| Cortical targets |  |  |  |
| Primary cortices | Primary motor cortex | Juelich microstructural atlas and  Harvard-Oxford cortical atlas | From Juelich: areas 46-49 |
|  | Primary somatosensory cortex |  | From Juelich: areas 50-57 |
|  | Primary visual cortex |  | From Juelich: areas 80-81 |
|  | Primary auditory cortex |  | From Juelich: Areas 40-45 |
| Associative cortices | Prefrontal associative cortex | Harvard-Oxford cortical atlas,  Faillenot et al. 2017 | frontal pole [1]  superior frontal gyrus [3]  middle frontal gyrus [4]  inferior frontal gyrus [5, 6]  frontal medial cortex [25]  orbitofrontal cortex [33]  paracingulate gyrus [28]  juxtapositional lobule cortex [26]  subcallosal cortex [27]  frontal operculum cortex [41] |
|  | Parietal associative cortex |  | superior parietal lobule [18]  supramarginal gyrus [19, 20]  angular gyrus [21]  precuneus cortex [31]  parietal operculum cortex [43]  central operculum cortex [42] |
|  | Occipital associative cortex |  | occipital fusiform gyrus [40]  lateral occipital cortex [22, 23]  Intracalcarine cortex [24]  cuneal cortex [32]  lingual gyrus [36]  occipital pole [48]  Supracalcarine cortex [47] |
|  | Temporal associative cortex |  | frontal pole [1]  superior frontal gyrus [3]  middle frontal gyrus [4]  inferior frontal gyrus [5, 6]  frontal medial cortex [25]  orbitofrontal cortex [33]  paracingulate gyrus [28]  juxtapositional lobule cortex [26]  subcallosal cortex [27]  frontal operculum cortex [41] |
|  | Anterior insular cortex |  | - |
|  | Posterior insular cortex |  | - |
|  | Anterior cingulate cortex |  | - |
|  | Posterior cingulate cortex |  | - |
|  | Hippocampus |  | - |
| Subcortical targets |  |  |  |
| Basal ganglia | Striatum | Harvard-Oxford subcortical | nucleus caudatus (cau)  putamen (put)  ncl. accumbens (na) |
|  | Pallidum |  | globus pallidus  ventral pallidum |
|  | Subthalamic nucleus |  | - |
| Neuromodulatory nuclei | Raphe nuclei | AAN | dorsal raphe (SC_NM_DR)  median raphe (SC_NM_MR) |
|  | Dopaminergic neuromodulatory nuclei | AAN, Pauli et al. 2018 | substantia nigra (SNc)  VTA |
|  | Cholinergic basal forebrain | Fritz et al. 2018 | ventral basal ganglia (ncl. accumbens, ventral pallidum)  Ncl. basalis  diagonal band of broca  substantia innominata  medial septal nucleus |
|  | Locus coeruleus | AAN | - |
| Other subcortical | Amygdala | Harvard-Oxford subcortical atlas | - |
|  | Thalamus |  | - |

Table S2: Incidental findings in the BLS cohort. All images were assessed by an experienced neuroradiologist after scanning. Incidental findings were noted in 22 out of the 81 included individuals. Abbreviations: DVA = developmental venous anomaly; LGG = Low grade glioma.

| N | Finding 1 | Finding 2 | Finding 3 |
| --- | --- | --- | --- |
| 1 | Pineal cyst 10 mm | - | - |
| 2 | Tornwaldt cyst | - | - |
| 3 | Megacisterna magna 1,8 cm x 4 cm | - | - |
| 4 | Ventricular enlargement | Frontal subcortical lesions (38) | Paratrigonal perivascular spaces |
| 5 | Pineal mass 13 mm | - | - |
| 6 | Pineal mass 11 mm | - | - |
| 7 | Tornwaldt cyst | - | - |
| 8 | Tornwaldt cyst | - | - |
| 9 | Arachnoidal cysts bilateral temporal poles | Meningeoma 9 mm left occipital pole | - |
| 10 | Left pulvinar Flair hyperintense lesion 11 mm x 7 mm, possible LGG | - | - |
| 11 | Pineal cyst 11 mm | - | - |
| 12 | Pineal mass 15 mm | - | - |
| 13 | Confluent white matter hyperintensity | - | - |
| 14 | Pituitary intermediate lobe cyst | - | - |
| 15 | Cerebellar lesion 9 mm | - | - |
| 16 | Pineal cyst 11 mm | - | - |
| 17 | DVA right cerebellum | - | - |
| 18 | Ventricular enlargement | - | - |
| 19 | Retrocerebellar arachnoidal cyst | - | - |
| 20 | Partial empty sella | - | - |
| 21 | Pineal mass 14 mm | - | - |
| 22 | Partial empty sella | - | - |

**Table S3: Streamline data for ipsilateral DWI-based connectivity of the claustrum.** See Table 2 for a list of abbreviations of the target regions. NOS is total number of reconstructed streamlines reaching the designated target region and corresponds to the “waytotal” output by FSL: **Abbreviations:** DWI = diffusion weighted imaging; FSL = FMRIB software library; CD = connection density; CP = connection probability; NOS = number of streamlines; ROI = region of interest.

| Target ROI | avg. target volume (mm^3^) |  | avg. NOS |  | avg. CP |  | avg. CD (mm^-3^) |  |
| --- | --- | --- | --- | --- | --- | --- | --- | --- |
| AC_Occ_R | 70344.73 | ± 6893.48 | 383207.56 | ± 112570.60 | 0.23 | ± 0.05 | 5.37 | ± 1.42 |
| AC_Occ_L | 71395.95 | ± 7164.69 | 406036.33 | ± 106474.07 | 0.27 | ± 0.05 | 5.59 | ± 1.32 |
| AC_Par_R | 64905.77 | ± 7072.67 | 497038.68 | ± 120733.81 | 0.30 | ± 0.05 | 7.65 | ± 1.97 |
| AC_Par_L | 64846.63 | ± 6986.11 | 331076.67 | ± 119012.44 | 0.21 | ± 0.05 | 5.06 | ± 1.75 |
| AC_PFC_R | 138013.90 | ± 14311.12 | 1072294.89 | ± 180019.63 | 0.64 | ± 0.05 | 7.76 | ± 1.23 |
| AC_PFC_L | 140720.59 | ± 13890.67 | 1028049.14 | ± 213323.65 | 0.67 | ± 0.05 | 7.25 | ± 1.36 |
| AC_Temp_R | 74539.89 | ± 7504.29 | 609099.51 | ± 138602.92 | 0.37 | ± 0.06 | 7.98 | ± 1.81 |
| AC_Temp_L | 72689.02 | ± 7420.18 | 527291.98 | ± 125353.76 | 0.35 | ± 0.07 | 7.28 | ± 1.87 |
| C_ant_R | 7041.14 | ± 1078.63 | 8302.35 | ± 6258.54 | 0.00 | ± 0.00 | 1.00 | ± 0.63 |
| C_ant_L | 7864.57 | ± 962.34 | 17414.81 | ± 15278.19 | 0.01 | ± 0.01 | 1.78 | ± 1.17 |
| C_pos_R | 7196.85 | ± 840.96 | 17219.68 | ± 7784.03 | 0.01 | ± 0.00 | 2.17 | ± 0.94 |
| C_pos_L | 7778.48 | ± 978.62 | 17913.02 | ± 9485.94 | 0.01 | ± 0.01 | 2.07 | ± 0.99 |
| Ins_ant_R | 3372.30 | ± 408.75 | 728321.58 | ± 144060.54 | 0.44 | ± 0.06 | 181.07 | ± 30.14 |
| Ins_ant_L | 3393.73 | ± 386.84 | 644091.16 | ± 166400.98 | 0.42 | ± 0.07 | 160.81 | ± 33.57 |
| Ins_pos_R | 4166.28 | ± 477.18 | 567714.64 | ± 139551.27 | 0.34 | ± 0.06 | 118.51 | ± 28.72 |
| Ins_pos_L | 3903.70 | ± 413.37 | 561487.26 | ± 138863.73 | 0.37 | ± 0.07 | 124.91 | ± 28.59 |
| PC_aud_R | 1402.17 | ± 237.67 | 46542.35 | ± 24259.58 | 0.03 | ± 0.01 | 21.69 | ± 8.69 |
| PC_aud_L | 1340.64 | ± 242.12 | 13946.43 | ± 9603.88 | 0.01 | ± 0.00 | 6.36 | ± 3.77 |
| PC_mot_R | 3848.70 | ± 577.39 | 10637.62 | ± 8928.38 | 0.01 | ± 0.00 | 2.24 | ± 1.72 |
| PC_mot_L | 4512.83 | ± 563.73 | 32878.30 | ± 15927.94 | 0.02 | ± 0.01 | 6.25 | ± 2.80 |
| PC_sens_R | 11903.72 | ± 1406.50 | 61679.93 | ± 32331.81 | 0.04 | ± 0.02 | 4.46 | ± 2.06 |
| PC_sens_L | 10750.05 | ± 1319.20 | 78624.07 | ± 33730.49 | 0.05 | ± 0.02 | 6.91 | ± 2.85 |
| PC_vis_R | 11453.86 | ± 1621.95 | 179273.37 | ± 63426.97 | 0.11 | ± 0.03 | 14.52 | ± 4.82 |
| PC_vis_L | 10101.11 | ± 1649.43 | 117736.49 | ± 38008.27 | 0.08 | ± 0.03 | 10.92 | ± 3.48 |
| SC_Amy_R | 2267.36 | ± 241.43 | 106325.54 | ± 49309.24 | 0.06 | ± 0.03 | 35.13 | ± 14.63 |
| SC_Amy_L | 1931.26 | ± 227.98 | 55869.16 | ± 31125.99 | 0.04 | ± 0.02 | 21.68 | ± 11.54 |
| SC_BG_Pal_R | 1827.93 | ± 199.53 | 12521.74 | ± 4670.56 | 0.01 | ± 0.00 | 4.83 | ± 1.46 |
| SC_BG_Pal_L | 1870.67 | ± 209.19 | 14748.60 | ± 6316.30 | 0.01 | ± 0.00 | 5.42 | ± 1.69 |
| SC_BG_SN_R | 48.62 | ± 12.71 | 668.37 | ± 396.25 | 0.00 | ± 0.00 | 0.82 | ± 0.31 |
| SC_BG_SN_L | 79.36 | ± 17.41 | 2403.75 | ± 1746.95 | 0.00 | ± 0.00 | 2.92 | ± 1.25 |
| SC_BG_Str_R | 8634.81 | ± 873.18 | 472298.99 | ± 137395.49 | 0.28 | ± 0.06 | 50.13 | ± 12.46 |
| SC_BG_Str_L | 8508.48 | ± 886.40 | 516613.01 | ± 161413.78 | 0.34 | ± 0.07 | 54.99 | ± 13.67 |
| SC_Hip_R | 4314.41 | ± 377.56 | 14302.16 | ± 7321.25 | 0.01 | ± 0.00 | 2.74 | ± 1.16 |
| SC_Hip_L | 4286.65 | ± 384.38 | 11458.12 | ± 4293.72 | 0.01 | ± 0.00 | 2.24 | ± 0.68 |
| SC_NM_cBF_R* | 2344.84 | ± 280.56 | 90841.53 | ± 58178.45 | 0.05 | ± 0.03 | 29.01 | ± 16.29 |
| SC_NM_cBF_L* | 2344.84 | ± 280.56 | 22648.22 | ± 10224.45 | 0.01 | ± 0.01 | 7.37 | ± 2.91 |
| SC_NM_Dop_R* | 274.07 | ± 40.12 | 1558.88 | ± 665.77 | 0.00 | ± 0.00 | 1.55 | ± 0.54 |
| SC_NM_Dop_L* | 274.07 | ± 40.12 | 2433.62 | ± 1484.72 | 0.00 | ± 0.00 | 2.37 | ± 0.98 |
| SC_NM_LC_R | 43.27 | ± 11.64 | 689.16 | ± 417.51 | 0.00 | ± 0.00 | 0.94 | ± 0.49 |
| SC_NM_LC_L | 43.44 | ± 13.42 | 1037.60 | ± 658.20 | 0.00 | ± 0.00 | 1.58 | ± 0.90 |
| SC_NM_R_R* | 419.17 | ± 61.57 | 1224.81 | ± 633.71 | 0.00 | ± 0.00 | 0.96 | ± 0.30 |
| SC_NM_R_L* | 419.17 | ± 61.57 | 1773.53 | ± 1016.41 | 0.00 | ± 0.00 | 1.61 | ± 0.72 |
| SC_Thal_R | 7581.04 | ± 755.94 | 34256.11 | ± 9711.38 | 0.02 | ± 0.01 | 4.18 | ± 1.18 |
| SC_Thal_L | 7716.74 | ± 782.60 | 47429.58 | ± 15966.16 | 0.03 | ± 0.01 | 5.74 | ± 1.91 |

**Table S4: Streamline data for contralateral DWI-based connectivity of the claustrum.** See Table 2 for a list of abbreviations of the target regions. NOS is total number of reconstructed streamlines reaching the designated target region and corresponds to the “waytotal” output by FSL: **Abbreviations:** DWI = diffusion weighted imaging; FSL = FMRIB software library; CD = connection density; CP = connection probability; NOS = number of streamlines; ROI = region of interest.

| Target ROI | avg. target volume (mm3) |  | avg. NOS |  | avg. CP | | avg. CD | |
| --- | --- | --- | --- | --- | --- | --- | --- | --- |
| AC_Occ_R | 70344.73 | ± 6893.48 | 20220.70 | ± 7644.00 | 0.01 | ± 0.00 | 0.29 | ± 0.11 |
| AC_Occ_L | 71395.95 | ± 7164.69 | 20582.00 | ± 7721.14 | 0.01 | ± 0.00 | 0.29 | ± 0.11 |
| AC_Par_R | 64905.77 | ± 7072.67 | 28622.20 | ± 11622.38 | 0.02 | ± 0.01 | 0.45 | ± 0.19 |
| AC_Par_L | 64846.63 | ± 6986.11 | 30690.36 | ± 10999.62 | 0.02 | ± 0.01 | 0.48 | ± 0.18 |
| AC_PFC_R | 138013.90 | ± 14311.12 | 110179.31 | ± 46788.12 | 0.07 | ± 0.03 | 0.80 | ± 0.33 |
| AC_PFC_L | 140720.59 | ± 13890.67 | 61454.07 | ± 29149.13 | 0.04 | ± 0.02 | 0.44 | ± 0.21 |
| AC_Temp_R | 74539.89 | ± 7504.29 | 6539.10 | ± 3035.59 | 0.00 | ± 0.00 | 0.09 | ± 0.04 |
| AC_Temp_L | 72689.02 | ± 7420.18 | 6308.83 | ± 3246.45 | 0.00 | ± 0.00 | 0.09 | ± 0.05 |
| C_ant_R | 7041.14 | ± 1078.63 | 26278.20 | ± 20450.33 | 0.02 | ± 0.01 | 3.43 | ± 2.32 |
| C_ant_L | 7864.57 | ± 962.34 | 4525.99 | ± 3959.02 | 0.00 | ± 0.00 | 0.54 | ± 0.48 |
| C_pos_R | 7196.85 | ± 840.96 | 12441.56 | ± 7309.12 | 0.01 | ± 0.00 | 1.61 | ± 0.92 |
| C_pos_L | 7778.48 | ± 978.62 | 10287.30 | ± 5256.00 | 0.01 | ± 0.00 | 1.23 | ± 0.61 |
| Ins_ant_R | 3372.30 | ± 408.75 | 595.69 | ± 513.45 | 0.00 | ± 0.00 | 0.15 | ± 0.13 |
| Ins_ant_L | 3393.73 | ± 386.84 | 689.05 | ± 512.82 | 0.00 | ± 0.00 | 0.17 | ± 0.14 |
| Ins_pos_R | 4166.28 | ± 477.18 | 365.49 | ± 311.83 | 0.00 | ± 0.00 | 0.79 | ± 0.70 |
| Ins_pos_L | 3903.70 | ± 413.37 | 643.04 | ± 565.91 | 0.00 | ± 0.00 | 0.14 | ± 0.13 |
| PC_aud_R | 1402.17 | ± 237.67 | 244.99 | ± 190.59 | 0.00 | ± 0.00 | 0.13 | ± 0.10 |
| PC_aud_L | 1340.64 | ± 242.12 | 225.44 | ± 169.57 | 0.00 | ± 0.00 | 0.12 | ± 0.10 |
| PC_mot_R | 3848.70 | ± 577.39 | 3135.04 | ± 3109.28 | 0.00 | ± 0.00 | 0.73 | ± 0.73 |
| PC_mot_L | 4512.83 | ± 563.73 | 2318.15 | ± 1791.65 | 0.00 | ± 0.00 | 0.46 | ± 0.37 |
| PC_sens_R | 11903.72 | ± 1406.50 | 7686.21 | ± 4736.75 | 0.01 | ± 0.00 | 0.63 | ± 0.39 |
| PC_sens_L | 10750.05 | ± 1319.20 | 6104.72 | ± 3156.58 | 0.00 | ± 0.00 | 0.54 | ± 0.29 |
| PC_vis_R | 11453.86 | ± 1621.95 | 13402.99 | ± 9525.15 | 0.01 | ± 0.01 | 1.13 | ± 0.81 |
| PC_vis_L | 10101.11 | ± 1649.43 | 12405.27 | ± 7102.94 | 0.01 | ± 0.00 | 1.18 | ± 0.71 |
| SC_Amy_R | 2267.36 | ± 241.43 | 3415.26 | ± 2192.20 | 0.00 | ± 0.00 | 1.21 | ± 0.81 |
| SC_Amy_L | 1931.26 | ± 227.98 | 1880.88 | ± 1746.58 | 0.00 | ± 0.00 | 0.73 | ± 0.65 |
| SC_BG_Pal_R | 1827.93 | ± 199.53 | 4275.14 | ± 3343.26 | 0.00 | ± 0.00 | 1.75 | ± 1.32 |
| SC_BG_Pal_L | 1870.67 | ± 209.19 | 2222.96 | ± 1285.48 | 0.00 | ± 0.00 | 0.88 | ± 0.50 |
| SC_BG_SN_R | 48.62 | ± 12.71 | 408.75 | ± 376.28 | 0.00 | ± 0.00 | 0.64 | ± 0.58 |
| SC_BG_SN_L | 79.36 | ± 17.41 | 261.89 | ± 176.30 | 0.00 | ± 0.00 | 0.36 | ± 0.25 |
| SC_BG_Str_R | 8634.81 | ± 873.18 | 13045.98 | ± 11799.56 | 0.01 | ± 0.01 | 1.40 | ± 1.24 |
| SC_BG_Str_L | 8508.48 | ± 886.40 | 8096.79 | ± 5904.62 | 0.00 | ± 0.00 | 0.88 | ± 0.66 |
| SC_Hip_R | 4314.41 | ± 377.56 | 2365.25 | ± 1625.44 | 0.00 | ± 0.00 | 0.49 | ± 0.35 |
| SC_Hip_L | 4286.65 | ± 384.38 | 1506.37 | ± 725.12 | 0.00 | ± 0.00 | 0.31 | ± 0.15 |
| SC_NM_LC_R | 43.27 | ± 11.64 | 531.98 | ± 536.82 | 0.00 | ± 0.00 | 0.84 | ± 0.89 |
| SC_NM_LC_L | 43.44 | ± 13.42 | 154.95 | ± 91.91 | 0.00 | ± 0.00 | 0.22 | ± 0.12 |
| SC_Thal_R | 7581.04 | ± 755.94 | 15040.16. | ± 13593.09 | 0.01 | ± 0.01 | 1.81 | ± 1.56 |
| SC_Thal_L | 7716.74 | ± 782.60 | 7639.30 | ± 3740.38 | 0.00 | ± 0.00 | 0.91 | ± 0.42 |

**Table S5: Correlation between CD and CP tract metrics for HCP cohort.** Information is shown for ipsilateral right side hemisphere tractography. See Table 2 for a list of abbreviations of the target regions. **Abbreviations:** CD = connection density; CP = connection probability; ROI = region of interest; Sig. = significance (p-value).

| Target ROI | avg. CP | avg. CD | Pearson correlation (r) | Sig. (2-tailed) |
| --- | --- | --- | --- | --- |
| AC_Occ | 0.2304 | 5.3745 | 0.768 | <.001 |
| AC_Par | 0.2967 | 7.6477 | 0.744 | <.001 |
| AC_PFC | 0.6432 | 7.7646 | 0.261 | 0.019 |
| AC_Temp | 0.3656 | 7.9842 | 0.755 | <.001 |
| C_ant | 0.0046 | 0.9957 | 0.935 | <.001 |
| C_pos | 0.0102 | 2.1717 | 0.929 | <.001 |
| Ins_ant | 0.4375 | 181.0693 | 0.634 | <.001 |
| Ins_pos | 0.3394 | 118.5082 | 0.771 | <.001 |
| PC_aud | 0.0264 | 21.6920 | 0.929 | <.001 |
| PC_mot | 0.0050 | 2.2402 | 0.957 | <.001 |
| PC_sens | 0.0350 | 4.4577 | 0.926 | <.001 |
| PC_vis | 0.1062 | 14.5173 | 0.851 | <.001 |
| SC_Amy | 0.0625 | 35.1256 | 0.948 | <.001 |
| SC_BG_Pal | 0.0072 | 4.8256 | 0.917 | <.001 |
| SC_BG_SN | 0.0003 | 0.8243 | 0.981 | <.001 |
| SC_BG_Str | 0.2812 | 50.1335 | 0.845 | <.001 |
| SC_Hip | 0.0082 | 2.7388 | 0.95 | <.001 |
| SC_NM_cBF | 0.0508 | 29.0119 | 0.952 | <.001 |
| SC_NM_Dop | 0.0008 | 1.5522 | 0.978 | <.001 |
| SC_NM_LC | 0.0003 | 0.9442 | 0.991 | <.001 |
| SC_NM_R | 0.0005 | 0.9626 | 0.884 | <.001 |
| SC_Thal | 0.0205 | 4.1784 | 0.847 | <.001 |

Table S6: Comparison of reconstructed ipsilateral streamline tracts in the two independent cohorts. See Table 2 for list of abbreviations of the target regions. Volumetric overlap of population average maps was calculated after thresholding at 10%. “overlap % BLS” represents the percentage of the thresholded BLS tract map volume that overlaps with the HCP reconstruction. and second. “overlap % HCP”. the percentage of the HCP tract map overlapping with the BLS tract map. Abbreviations: ROI = region of interest; BLS = Bavarian longitudinal study; HCP = human connectome project; vol. = volume. *all volumes in mm^3^.

| Target ROI | BLS tract vol.* | HCP tract vol.* | Overlap vol.* | overlap % BLS | overlap % HCP |
| --- | --- | --- | --- | --- | --- |
| AC_Occ_R | 60992 | 26568 | 24840 | 40.7% | 93.5% |
| AC_Occ_L | 33232 | 14560 | 13624 | 41.0% | 93.6% |
| AC_Par_R | 26592 | 7768 | 7504 | 28.2% | 96.6% |
| AC_Par_L | 24176 | 8856 | 8376 | 34.6% | 94.6% |
| AC_PFC_R | 21944 | 8208 | 7896 | 36.0% | 96.2% |
| AC_PFC_L | 22688 | 8920 | 8600 | 37.9% | 96.4% |
| AC_Temp_R | 18872 | 8872 | 7664 | 40.6% | 86.4% |
| AC_Temp_L | 18792 | 8184 | 7752 | 41.3% | 94.7% |
| C_ant_R | 23312 | 14584 | 12504 | 53.6% | 85.7% |
| C_ant_L | 24072 | 14552 | 12544 | 52.1% | 86.2% |
| C_pos_R | 29168 | 18600 | 17448 | 59.8% | 93.8% |
| C_pos_L | 32344 | 24816 | 19192 | 59.3% | 77.3% |
| Ins_ant_R | 5032 | 2792 | 2712 | 53.9% | 97.1% |
| Ins_ant_L | 4968 | 2712 | 2664 | 53.6% | 98.2% |
| Ins_pos_R | 5304 | 2536 | 2488 | 46.9% | 98.1% |
| Ins_pos_L | 4704 | 2656 | 2576 | 54.8% | 97.0% |
| PC_aud_R | 16744 | 9520 | 9008 | 53.8% | 94.6% |
| PC_aud_L | 16216 | 10336 | 9168 | 56.5% | 88.7% |
| PC_mot_R | 19792 | 13184 | 11768 | 59.5% | 89.3% |
| PC_mot_L | 19824 | 12040 | 10512 | 53.0% | 87.3% |
| PC_sens_R | 18336 | 11560 | 10800 | 58.9% | 93.4% |
| PC_sens_L | 18704 | 10792 | 9992 | 53.4% | 92.6% |
| PC_vis_R | 22104 | 13640 | 13120 | 59.4% | 96.2% |
| PC_vis_L | 28616 | 15688 | 14928 | 52.2% | 95.2% |
| SC_Amy_R | 3496 | 1824 | 1088 | 31.1% | 59.6% |
| SC_Amy_L | 4328 | 3568 | 2488 | 57.5% | 69.7% |
| SC_BG_Pal_R | 25840 | 18360 | 12424 | 48.1% | 67.7% |
| SC_BG_Pal_L | 32128 | 14640 | 12592 | 39.2% | 86.0% |
| SC_BG_SN_R | 30088 | 19544 | 15872 | 52.8% | 81.2% |
| SC_BG_SN_L | 26592 | 13608 | 12408 | 46.7% | 91.2% |
| SC_BG_Str_R | 5576 | 3128 | 3096 | 55.5% | 99.0% |
| SC_BG_Str_L | 5288 | 3088 | 3064 | 57.9% | 99.2% |
| SC_Hip_R | 34912 | 12552 | 10832 | 31.0% | 86.3% |
| SC_Hip_L | 42256 | 19920 | 17528 | 41.5% | 88.0% |
| SC_NM_cBF_R | 5544 | 3184 | 1912 | 34.5% | 60.1% |
| SC_NM_cBF_L | 17272 | 9648 | 6864 | 39.7% | 71.1% |
| SC_NM_Dop_R | 39704 | 21240 | 17296 | 43.6% | 81.4% |
| SC_NM_Dop_L | 41104 | 19056 | 16272 | 39.6% | 85.4% |
| SC_NM_LC_R | 44896 | 27936 | 22584 | 54.4% | 80.8% |
| SC_NM_LC_L | 44896 | 20216 | 18920 | 42.1% | 93.6% |
| SC_NM_R_R | 50088 | 24080 | 20752 | 41.4% | 86.2% |
| SC_NM_R_L | 60184 | 21976 | 19144 | 31.8% | 87.1% |
| SC_Thal_R | 32488 | 17520 | 15032 | 46.3% | 85.8% |
| SC_Thal_L | 36168 | 17816 | 15576 | 43.1% | 87.4% |

**Table S7: Voxel-wise correlation values between HCP and BLS cohorts on cohort-averaged tract maps.** Tract maps were thresholded at 10% (0.1). **Abbreviations**: BLS = Bavarian longitudinal study; HCP = human connectome project; ROI = region of interest.

| **target ROI** | **Left side** | **Right side** |
| --- | --- | --- |
| **AC_Occ** | 0.80 | 0.79 |
| **AC_Par** | 0.73 | 0.64 |
| **AC_PFC** | 0.79 | 0.80 |
| **AC_Temp** | 0.80 | 0.74 |
| **C_ant** | 0.83 | 0.81 |
| **C_pos** | 0.77 | 0.83 |
| **PC_aud** | 0.78 | 0.81 |
| **PC_mot** | 0.79 | 0.85 |
| **PC_sens** | 0.81 | 0.87 |
| **PC_vis** | 0.86 | 0.92 |
| **SC_BG_Pal** | 0.68 | 0.64 |
| **SC_BG_SN** | 0.79 | 0.83 |
| **SC_Hip** | 0.71 | 0.70 |
| **SC_NM_Dop** | 0.72 | 0.76 |
| **SC_NM_LC** | 0.75 | 0.79 |
| **SC_NM_R** | 0.72 | 0.79 |
| **SC_Thal** | 0.71 | 0.69 |
| **Means** | **0.77 ± 0.05** | **0.78 ± 0.08** |

**Table S8: Voxel-wise average correlation values between 6561 subject pairs within and across cohorts**. Individual subject’s streamline reconstructions were thresholded at 1% (0.01) and normalized to MNI space. **Abbreviations**: BLS = Bavarian longitudinal study; HCP = human connectome project; ROI = region of interest.

| **target ROI** | **Left side** | **Right side** | **Left side HCP** | **Right side HCP** | **Left side BLS** | **Right side BLS** |
| --- | --- | --- | --- | --- | --- | --- |
| **AC_Occ** | 0.462 | 0.462 | 0.589 | 0.599 | 0.473 | 0.484 |
| **AC_Par** | 0.328 | 0.307 | 0.416 | 0.451 | 0.384 | 0.431 |
| **AC_PFC** | 0.425 | 0.468 | 0.479 | 0.524 | 0.486 | 0.508 |
| **AC_Temp** | 0.354 | 0.355 | 0.462 | 0.412 | 0.466 | 0.491 |
| **C_ant** | 0.238 | 0.237 | 0.254 | 0.249 | 0.286 | 0.299 |
| **C_pos** | 0.246 | 0.291 | 0.273 | 0.325 | 0.366 | 0.359 |
| **PC_aud** | 0.222 | 0.231 | 0.291 | 0.319 | 0.264 | 0.256 |
| **PC_mot** | 0.272 | 0.298 | 0.366 | 0.338 | 0.340 | 0.389 |
| **PC_sens** | 0.337 | 0.370 | 0.463 | 0.428 | 0.411 | 0.431 |
| **PC_vis** | 0.412 | 0.476 | 0.520 | 0.577 | 0.413 | 0.458 |
| **SC_BG_Pal** | 0.198 | 0.198 | 0.243 | 0.283 | 0.281 | 0.285 |
| **SC_BG_SN** | 0.285 | 0.276 | 0.329 | 0.305 | 0.349 | 0.316 |
| **SC_Hip** | 0.208 | 0.261 | 0.273 | 0.383 | 0.271 | 0.261 |
| **SC_NM_Dop** | 0.204 | 0.246 | 0.258 | 0.307 | 0.305 | 0.324 |
| **SC_NM_LC** | 0.283 | 0.284 | 0.399 | 0.376 | 0.329 | 0.318 |
| **SC_NM_R** | 0.235 | 0.288 | 0.308 | 0.353 | 0.290 | 0.330 |
| **SC_Thal** | 0.232 | 0.232 | 0.294 | 0.371 | 0.406 | 0.358 |
| **Means** | **0.291 ± 0.083** | **0.311 ± 0.087** | **0.366 ± 0.106** | **0.388 ± 0.100** | **0.360 ± 0.073** | **0.370 ± 0.083** |

**Table S9: Cohort-average contralateral tract atlas comparison between datasets using dice coefficient (DC).** DC was calculated for each target region between corresponding tracts in HCP and BLS cohorts using cohort-averaged tracts thresholded at 10% (0.1). Please refer to Table 4 in main manuscript for data on ipsilateral connectivity. **Abbreviations**: BLS = Bavarian longitudinal study; DC = dice coefficient; HCP = human connectome project; ROI = region of interest.

| **target ROI** | **DC left side** | **DC right side** |
| --- | --- | --- |
| **AC_Occ** | 0.581 | 0.567 |
| **AC_Par** | 0.578 | 0.560 |
| **AC_PFC** | 0.431 | 0.396 |
| **AC_Temp** | 0.550 | 0.603 |
| **C_ant** | 0.634 | 0.238 |
| **C_pos** | 0.706 | 0.731 |
| **Ins_ant** | 0.595 | 0.531 |
| **Ins_pos** | 0.656 | 0.633 |
| **PC_aud** | 0.665 | 0.536 |
| **PC_mot** | 0.512 | 0.558 |
| **PC_sens** | 0.624 | 0.678 |
| **PC_vis** | 0.498 | 0.472 |
| **SC_Amy** | 0.465 | 0.587 |
| **SC_BG_Pal** | 0.456 | 0.459 |
| **SC_BG_SN** | 0.724 | 0.658 |
| **SC_BG_Str** | 0.391 | 0.383 |
| **SC_Hip** | 0.494 | 0.572 |
| **SC_NM_LC** | 0.545 | 0.483 |
| **SC_Thal** | 0.550 | 0.564 |
| **Means** | **0.561 ± 0.093** | **0.537 ± 0.115** |

Table S10: Control analysis on the influence of insula and putamen connectivity on claustrum CD across target ROIs, HCP cohort. The model containing connectivity from both the insula and the putamen explained 44.5% of the variance (avg. R² = .445, avg. Adjusted R² = .431). Data is shown for right side ipsilateral connectivity on HCP cohort, see main manuscript for left side connectivity (Table 5). Abbreviations: HCP = human connectome project; ROI = region of interest; St. dev. = standard deviation.

| *target ROI* | t-test |  | multiple regression analysis | | | | | | | | | |
| --- | --- | --- | --- | --- | --- | --- | --- | --- | --- | --- | --- | --- |
|  | t | p | **Putamen** | | | | **Insula** | | | | R^2^ | adj. R^2^ |
|  |  |  | B | Beta | t | p | B | Beta | t | p |  |  |
| AC_Occ | 34.18 | <.001 | .017 | .042 | .392 | .696 | .179 | .401 | 3.76 | <.001 | .171 | .149 |
| AC_Par | 34.93 | <.001 | .062 | .184 | 1.686 | .096 | .046 | .251 | 2.30 | .024 | .118 | .096 |
| AC_PFC | 56.59 | <.001 | .034 | .219 | 2.097 | .039 | .101 | .329 | 3.15 | .002 | .186 | .165 |
| AC_Temp | 25.28 | <.001 | .308 | .299 | 2.901 | .005 | .068 | .344 | 3.33 | .001 | .183 | .162 |
| C_ant | 12.57 | <.001 | .001 | .014 | .132 | .895 | .413 | .695 | 6.66 | <.001 | .496 | .483 |
| C_pos | 20.07 | <.001 | .048 | .322 | 3.486 | <.001 | .180 | .504 | 5.46 | <.001 | .536 | .524 |
| PC_aud | 18.88 | <.001 | 3.21 | .381 | 3.822 | <.001 | .085 | .267 | 2.68 | .009 | .225 | .205 |
| PC_mot | 10.19 | <.001 | -.013 | -.117 | -1.87 | .065 | .492 | .861 | 13.8 | <.001 | .710 | .703 |
| PC_sens | 16.45 | <.001 | .005 | .023 | .302 | .763 | .234 | .743 | 9.74 | <.001 | .549 | .538 |
| PC_vis | 25.11 | <.001 | .051 | .072 | .740 | .461 | .581 | .522 | 5.35 | <.001 | .295 | .277 |
| SC_BG_Pal | 25.08 | <.001 | - | - | - | - | .217 | .710 | 8.95 | <.001 | .504 | .497 |
| SC_BG_SN | 15.34 | <.001 | .047 | .265 | 3,426 | <.001 | .622 | .650 | 8.40 | <.001 | .542 | .531 |
| SC_Hip | 17.53 | <.001 | .020 | .066 | .582 | .563 | .395 | .350 | 3.10 | .003 | .144 | .122 |
| SC_NM_Dop | 21.41 | <.001 | .026 | .249 | 4.200 | <.001 | .455 | .765 | 12.9 | <.001 | .743 | .736 |
| SC_NM_LC | 15.57 | <.001 | .056 | .237 | 3.991 | <.001 | .944 | .758 | 12.8 | <.001 | .761 | .755 |
| SC_NM_R | 17.01 | <.001 | .031 | .198 | 4.004 | <.001 | .574 | .831 | 16.8 | <.001 | .825 | .820 |
| SC_Thal | 31.94 | <.001 | .012 | .152 | 1.944 | .056 | .162 | 696 | 8.92 | <.001 | .582 | .571 |
| Means |  |  |  |  |  |  |  |  |  |  | **.445** | **.431** |
| St. dev. |  |  |  |  |  |  |  |  |  |  | **.235** | **.241** |

Table S11: Control analysis on the influence of insula and putamen connectivity on claustrum CP across target ROIs, HCP cohort. The model containing connectivity from both the insula and the putamen explained 46.9% of the variance (avg. R² = .469, avg. Adjusted R² = .455). Data is shown for right side ipsilateral connectivity on HCP cohort, see main manuscript for left side connectivity (Table 6). Abbreviations: HCP = human connectome project; ROI = region of interest; St. dev. = standard deviation.

| target ROI | t-test |  | multiple regression analysis | | | | | | | | | |
| --- | --- | --- | --- | --- | --- | --- | --- | --- | --- | --- | --- | --- |
|  | t | p | **Putamen** | | | | **Insula** | | | | R^2^ | adj. R^2^ |
|  |  |  | B | Beta | t | p | B | Beta | t | p |  |  |
| AC_Occ | 37.80 | <.001 | .101 | .036 | .344 | .731 | 1.97 | .429 | 4.14 | <.001 | .191 | .170 |
| AC_Par | 52.47 | <.001 | .286 | .173 | 1.66 | .101 | .520 | .347 | 3.33 | .001 | .168 | .147 |
| AC_PFC | 109.8 | <.001 | .191 | .311 | 2.95 | .004 | .434 | .188 | 1.79 | .007 | .139 | .116 |
| AC_Temp | 51.13 | <.001 | 1.20 | .252 | 2.48 | .015 | .639 | .405 | 3.98 | <.001 | .204 | .184 |
| C_ant | 12.75 | <.001 | .003 | .008 | .084 | .933 | 2.60 | .746 | 7.68 | <.001 | .565 | .554 |
| C_pos | 22.23 | <.001 | .155 | .237 | 2.73 | .008 | 1.19 | .613 | 7.05 | <.001 | .595 | .585 |
| PC_aud | 20.38 | <.001 | 7.78 | .418 | 4.44 | <.001 | .259 | .343 | 3.65 | <.001 | .312 | .294 |
| PC_mot | 10.50 | <.001 | -.047 | .023 | -.114 | .044 | 2.23 | .897 | 16.2 | <.001 | .772 | .767 |
| PC_sens | 17.41 | <.001 | .011 | .010 | .149 | .882 | 1.78 | .809 | 12.1 | <.001 | .653 | .644 |
| PC_vis | 27.40 | <.001 | .221 | .057 | .570 | .570 | 3.88 | .475 | 4.75 | <.001 | .237 | .218 |
| SC_BG_Pal | 26.22 | <.001 | - | - | - | - | .674 | .754 | 10.2 | <.001 | .568 | .563 |
| SC_BG_SN | 12.92 | <.001 | .049 | .249 | 3.18 | .002 | .691 | .646 | 8.23 | <.001 | .531 | .519 |
| SC_Hip | 18.12 | <.001 | .044 | .037 | .318 | .751 | 1.63 | .312 | 2.69 | .009 | .107 | .085 |
| SC_NM_Dop | 19.30 | <.001 | .039 | .260 | 4.61 | <.001 | .664 | .773 | 13.7 | <.001 | .767 | .761 |
| SC_NM_LC | 13.78 | <.001 | .060 | .238 | 3.98 | <.001 | .974 | .755 | 12.6 | <.001 | .758 | .752 |
| SC_NM_R | 15.92 | <.001 | .051 | .216 | 4.25 | <.001 | .885 | .817 | 14.1 | <.001 | .814 | .810 |
| SC_Thal | 35.66 | <.001 | .047 | .128 | 1.65 | .103 | .974 | .713 | 9.22 | <.001 | .585 | .574 |
| Means |  |  |  |  |  |  |  |  |  |  | **.469** | **.455** |
| St. dev. |  |  |  |  |  |  |  |  |  |  | **.246** | **.252** |

Table S12: Control analysis on the influence of insula and putamen connectivity on claustrum CD across target ROIs, BLS cohort. The model containing connectivity from both the insula and the putamen explained 48.5 % of the variance (avg. R² = .485, avg. Adjusted R² = .473). Data is shown for right side ipsilateral connectivity on BLS cohort, see main manuscript for left side connectivity (Table 7). Abbreviations: BLS = Bavarian longitunial study; ROI = region of interest; St. dev. = standard deviation.

| *target ROI* | t-test |  | multiple regression analysis | | | | | | | | | |
| --- | --- | --- | --- | --- | --- | --- | --- | --- | --- | --- | --- | --- |
|  | t | p | **Putamen** | | | | **Insula** | | | | R^2^ | adj. R^2^ |
|  |  |  | B | Beta | t | p | B | Beta | t | p |  |  |
| AC_Occ | 20.61 | <.001 | .094 | .265 | 2.62 | .010 | .215 | .439 | 4.35 | <.001 | .369 | .353 |
| AC_Par | 25.89 | <.001 | .037 | .156 | 1.51 | .136 | .062 | .388 | 3.75 | <.001 | .197 | .177 |
| AC_PFC | 49.05 | <.001 | .044 | .249 | 2.24 | .028 | .021 | .075 | .677 | .500 | .075 | .051 |
| AC_Temp | 29.39 | <.001 | .255 | .335 | 3.31 | .001 | .068 | .329 | 3.25 | .002 | .297 | .279 |
| C_ant | 8.03 | <.001 | .068 | .305 | 5.47 | <.001 | .952 | .708 | 12.7 | <.001 | .830 | .825 |
| C_pos | 8.71 | <.001 | .139 | .399 | 4.99 | <.001 | .335 | .548 | 6.86 | <.001 | .795 | .790 |
| PC_aud | 8.78 | <.001 | 1.13 | .474 | 5.17 | <.001 | .041 | .309 | 3.38 | .001 | .352 | .335 |
| PC_mot | 10.10 | <.001 | .033 | .208 | 3.56 | <.001 | .576 | .785 | 13.5 | <.001 | .760 | .754 |
| PC_sens | 14.58 | <.001 | .038 | .233 | 3.27 | .002 | .286 | .715 | 10.0 | <.001 | .611 | .601 |
| PC_vis | 10.45 | <.001 | .156 | .149 | 1.35 | .183 | .810 | .475 | 4.27 | <.001 | .325 | .308 |
| SC_BG_Pal | 10.53 | <.001 | - | - | - | - | .814 | .434 | 4.28 | <.001 | .188 | .178 |
| SC_BG_SN | 11.04 | <.001 | .054 | .281 | 3.40 | .001 | 1.62 | .582 | 7.04 | <.001 | .513 | .500 |
| SC_Hip | 5.86 | <.001 | .035 | .065 | .643 | .522 | 1.33 | .562 | 5.54 | <.001 | .352 | .336 |
| SC_NM_Dop | 12.86 | <.001 | .031 | .233 | 2.94 | .004 | 1.47 | .646 | 8.14 | <.001 | .540 | .528 |
| SC_NM_LC | 10.03 | <.001 | .100 | .365 | 5.34 | <.001 | 1.92 | .607 | 8.88 | <.001 | .768 | .762 |
| SC_NM_R | 11.28 | <.001 | .053 | .485 | 5.55 | <.001 | .849 | .395 | 4.52 | <.001 | .620 | .610 |
| SC_Thal | 14.98 | <.001 | .057 | .291 | 3.70 | <.001 | .427 | .615 | 7.82 | <.001 | .657 | .649 |
| Means |  |  |  |  |  |  |  |  |  |  | **.485** | **.473** |
| St. dev. |  |  |  |  |  |  |  |  |  |  | **.236** | **.241** |

Table S13: Control analysis on the influence of insula and putamen connectivity on claustrum CP across target ROIs, BLS cohort. The model containing connectivity from both the insula and the putamen explained 49.7% of the variance (avg. R² = .497, avg. Adjusted R² = .485). Data is shown for right side ipsilateral connectivity on BLS cohort, see main manuscript for left side connectivity (Table 8). Abbreviations: BLS = Bavarian longitunial study; ROI = region of interest; St. dev. = standard deviation.

| target ROI | t-test |  | multiple regression analysis | | | | | | | | | |
| --- | --- | --- | --- | --- | --- | --- | --- | --- | --- | --- | --- | --- |
|  | t | p | **Putamen** | | | | **Insula** | | | | R^2^ | adj. R^2^ |
|  |  |  | B | Beta | t | p | B | Beta | t | p |  |  |
| AC_Occ | 22.72 | <.001 | .635 | .272 | 2.95 | .004 | 2.42 | .518 | 5.61 | <.001 | .471 | .457 |
| AC_Par | 34.22 | <.001 | .270 | .207 | 1.99 | .050 | .464 | .357 | 3.44 | <.001 | .202 | .182 |
| AC_PFC | 82.77 | <.001 | .453 | .547 | 5.96 | <.001 | .371 | .172 | 1.88 | .064 | .349 | .333 |
| AC_Temp | 35.64 | <.001 | 1.33 | .288 | 2.93 | .005 | .724 | .394 | 4.00 | <.001 | .301 | .283 |
| C_ant | 8.33 | <.001 | .287 | .260 | 4.03 | <.001 | 5.49 | .709 | 11.0 | <.001 | .775 | .769 |
| C_pos | 8.09 | <.001 | .837 | .455 | 6.70 | <.001 | 2.12 | .527 | 7.76 | <.001 | .849 | .845 |
| PC_aud | 8.90 | <.001 | 3.06 | .537 | 6.30 | <.001 | .112 | .331 | 3.88 | <.001 | .441 | .427 |
| PC_mot | 10.44 | <.001 | .026 | .089 | 1.37 | .174 | 2.61 | .817 | 12.6 | <.001 | .672 | .664 |
| PC_sens | 15.55 | <.001 | .194 | .216 | 2.90 | .005 | 1.92 | .696 | 9.33 | <.001 | .575 | .564 |
| PC_vis | 9.99 | <.001 | .778 | .116 | 1.18 | .244 | 7.72 | .600 | 6.07 | <.001 | .447 | .432 |
| SC_BG_Pal | 9.86 | <.001 | - | - | - | - | 2.55 | .422 | 4.14 | <.001 | .178 | .168 |
| SC_BG_SN | 8.78 | <.001 | .065 | .314 | 3.56 | <.001 | 1.52 | .505 | 5.73 | <.001 | .446 | .431 |
| SC_Hip | 6.25 | <.001 | .219 | .105 | 1.05 | .295 | 6.30 | .568 | 5.70 | <.001 | .388 | .372 |
| SC_NM_Dop | 12.14 | <.001 | .039 | .220 | 2.60 | .011 | 1.88 | .607 | 7.18 | <.001 | .486 | .473 |
| SC_NM_LC | 8.00 | <.001 | .133 | .463 | 6.35 | <.001 | 1.68 | .495 | 6.79 | <.001 | .707 | .699 |
| SC_NM_R | 9.60 | <.001 | .080 | .474 | 5.19 | <.001 | 1.18 | .372 | 4.07 | <.001 | .563 | .552 |
| SC_Thal | 14.53 | <.001 | .353 | .334 | 3.89 | <.001 | 2.49 | .539 | 6.26 | <.002 | .603 | .592 |
| Means |  |  |  |  |  |  |  |  |  |  | **.497** | **.485** |
| St. dev. |  |  |  |  |  |  |  |  |  |  | **.188** | **.192** |
